# Protein *O*-mannosylation deficiency increases LprG-associated lipoarabinomannan release by *Mycobacterium tuberculosis* and enhances the TLR2-associated inflammatory response

**DOI:** 10.1038/s41598-017-08489-7

**Published:** 2017-08-11

**Authors:** Henar Alonso, Julien Parra, Wladimir Malaga, Delphine Payros, Chia-Fang Liu, Céline Berrone, Camille Robert, Etienne Meunier, Odile Burlet-Schiltz, Michel Rivière, Christophe Guilhot

**Affiliations:** Institut de Pharmacologie et de Biologie Structurale, IPBS, Université de Toulouse, CNRS, UPS, Toulouse, France

## Abstract

Protein *O*-mannosylation is crucial for the biology of *Mycobacterium tuberculosis* but the key mannosylated protein(s) involved and its(their) underlying function(s) remain unknown. Here, we demonstrated that the *M*. *tuberculosis* mutant (Δ*pmt*) deficient for protein *O*-mannosylation exhibits enhanced release of lipoarabinomannan (LAM) in a complex with LprG, a lipoprotein required for LAM translocation to the cell surface. We determined that LprG is *O-*mannosylated at a unique threonine position by mass spectrometry analyses of the purified protein. However, although replacement of this amino acid by an alanine residue completely abolished LprG *O-*mannosylation, the increased release of the LAM/LprG complex was preserved. We found that the increased secretion of this complex is due to enhanced LAM production in the Δ*pmt M*. *tuberculosis* and *M*. *smegmatis* mutants relative to their wild-type counterparts. This abnormal release of LAM/LprG has functional consequences on the induction of inflammatory responses and provides a possible explanation for the reduced virulence of the *M*. *tuberculosis* Δ*pmt* mutant.

## Introduction

Glycosylation is one of the most common post-translational modifications of proteins that considerably affects protein properties/functions and biological activities in eukaryotes. Until recently, protein glycosylation was thought to occur only rarely in prokaryotes with limited impact on their biology^1^. However, the analysis of protein reactivity with the lectin Concanavalin A and the development of mass spectrometry (MS) for protein analysis have revealed that several proteins in actinomycetes are *O-*mannosylated, especially in the human major pathogen, *Mycobacterium tuberculosis*. The first identified glycoproteins were the secreted antigens PstS1 (38 kDa), the alanine-proline rich Apa protein (45–47 kDa) and the lipoprotein LpqH (19 kDa)^2–5^. These results garnered immediate widespread attention because this post-translational modification may play an important role in the biology of *M*. *tuberculosis* and the host/pathogen interaction. Indeed, *M*. *tuberculosis* is the causative agent of human tuberculosis (TB), a disease which still remains a major public health concern with 10.4 million new cases and 1.8 million deaths in 2015^6^. A better understanding of the strategies used by this pathogen to circumvent the host immune defense, and thus to persist and to cause disease, may reveal weak spots in the TB bacilli that can be exploited to develop innovative approaches to control this important pathogen and the associated disease.

*O-*mannosylated proteins purified from mycobacteria bind to host innate-immune receptors, such as lung surfactant protein A, DC-SIGN (dendritic-cell-specific intercellular molecule-3-grabbing non-integrin), and the mannose receptor and contribute to phagocyte invasion^7–9^. In addition, T cell antigenicity of secreted *M*. *tuberculosis* glycoproteins is modulated by the pattern of *O-*mannosylation. The best documented example is the Apa antigen, which contains up to nine mannose residues when purified from *M*. *tuberculosis* or the model mycobacterial strain *Mycobacterium smegmatis*, but no sugar residue when expressed in, and purified from, *Escherichia coli* ^10, 11^. The glycosylated forms of Apa induce strong delayed-type hypersensitivity reactions (DTH) *in vivo* and exhibit a high capacity to stimulate T lymphocyte responses of guinea pigs immunized with BCG. In contrast, non-glycosylated Apa, obtained either following deglycosylation of the native form from *M*. *tuberculosis* or purified from *E*. *coli*, is more than 10-fold less potent in inducing these responses^10, 11^. Similar results have been obtained in mice and humans showing that *O-*mannosylation of Apa enhances its T cell antigenicity^12^. However, glycosylation of this antigen is dispensable for the induction of a protective immune response in mice^12^.

The key finding demonstrating the major role played by *O-*mannosylation in the biology of TB bacilli came from the construction and the phenotypic characterization of a defined *M*. *tuberculosis* mutant deficient for this post-translational modification^13^. VanderVen *et al*.^14^ identified a protein, encoded by the *Rv1002c* gene in *M*. *tuberculosis*, that exhibits sequence similarities with membrane-associated protein *O*-mannosyl transferase from *Saccharomyces cerevisiae*. They demonstrated that *Rv1002c* (*pmt*) encodes an *O*-mannosyl transferase that catalyzes the initial step of protein glycosylation in *M*. *tuberculosis* and that this process is coupled to Sec-translocation^14^. Knock-out of *Rv1002c*, and its ortholog *Msmeg_5447*, in *M*. *tuberculosis* and *M*. *smegmatis* respectively demonstrated that these genes are dispensable for *in vitro* mycobacterial growth, in contrast to eukaryotes where protein *O*-mannosylation is required for viability^13^. Mass-spectrometry (MS) analyses showed that the mutant strains were indeed deficient in protein *O*-mannosylation consistent with the hypothesis that *Rv1002*c and *MSMEG_5447* have no paralogs in *M*. *tuberculosis* and *M*. *smegmatis* respectively. The *M*. *tuberculosis* Δ*Rv1002c::km* (Δ*pmt*) mutant displayed several phenotypes *in vitro* and *in vivo* demonstrating that protein *O*-mannosylation is a major post-translational modification for the biology of this pathogen. First, the ability of the Δ*pmt* mutant to form colonies on solid medium was profoundly impaired and it exhibited delayed growth in liquid medium containing dextrose as the sole carbon source. Second, this mutant was strongly attenuated in several cellular and animal infection models: its growth in mouse alveolar macrophages or primary human blood monocyte-derived macrophages and its capacity to kill immunocompromised mice were severely impaired^13^. These results established Rv1002c as a membrane-associated protein *O*-mannosyl transferase of crucial importance for the biology of *M*. *tuberculosis* and especially for its interaction with the host. However, these studies did not identify the Rv1002c-target proteins responsible for the *in vitro* growth defect and attenuated phenotype when not glycosylated.

Two main strategies have been used to identify glycosylated proteins in *M*. *tuberculosis*. Zamorano *et al*.^15^ performed glycoproteins enrichment using the lectin Concanavalin A, followed by 2D gel electrophoresis and conventional mass spectrometry analysis of the individual spots. They identified 41 proteins, but did not provide direct evidence that they were indeed glycosylated. More recently, advances in MS technologies have allowed the development of analytical approaches to directly search for glycopeptides in complex protein extracts. For example, Smith *et al*.^16^ used liquid chromatography (LC) coupled to tandem MS analyses to identify 14 glycopeptides, and to localize their glycosylsation sites, in *M*. *tuberculosis* culture filtrate corresponding to 13 glycosylated proteins. These results largely extended the repertoire of *M*. *tuberculosis* glycoproteins but did not address the question of the impact of this post-translational modification on the functions of these proteins.

Here, we aimed to address this question and to examine how the *O-*mannosylation of *M*. *tuberculosis* proteins may contribute to host/pathogen interactions. We identified the lipoprotein, LprG, as a new *O*-mannosylated protein and characterized its glycosylation site. We demonstrated that the lack of protein *O*-mannosylation is associated with increased production of lipoarabinomannnan (LAM), a critical mycobacterial virulence factor^17^, and increased release of this lypoglycan complexed with LprG. We found that this perturbation is associated with enhanced stimulation of Toll-Like Receptor 2 (TLR2), a key host receptor to control *M*. *tuberculosis* infection^18^, and a stronger induction of inflammatory responses by infected macrophages.

## Results

### Phenotypes of M. tuberculosis mutants deficient in selected mannoprotein production

**Figure 1:**
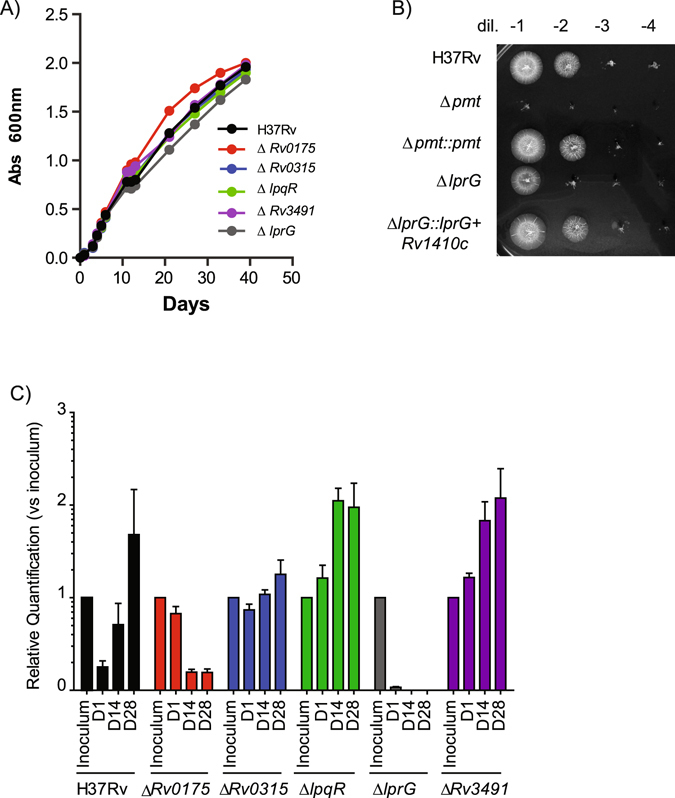
Contribution of selected mannoproteins to *M*. *tuberculosis* growth *in vitro* and *in vivo*. (**A**) Growth of WT *M*. *tuberculosis* and mutants in liquid medium. Mycobacterial strains were inoculated at 10^6^cfu/ml. Absorbance at 600 nm was followed over time for the various cultures. (**B**) Growth of WT *M*. *tuberculosis* and mutants on 7H11 Petri plates. Serial dilutions of adjusted liquid cultures of the various mutants were spotted on solid medium and incubated at 37 °C. (**C**) Growth of tagged-WT *M*. *tuberculosis* and tagged-mutants in BALB/c mice. Mice (5 for each time points) were infected with 6.10^2^ cfu of a population containing similar concentration of each strains. At 1, 14 or 28 days post-infection, the bacterial population was recovered from the lung of the infected mice and grown for 7 days in liquid medium. Total DNA was extracted and the quantity of each mutant evaluated by qPCR using primers specific for the tags. The amount of each mutant relative to the inoculum is plotted.

We applied the recently developed glycopeptidomics approach^13^ for the analysis of the *M*. *tuberculosis* secretome to further enrich the mannoprotein repertoire of *M*. *tuberculosis*. The combination of high efficiency SDS-PAGE based shotgun MS proteomics and automated post-analytical data mining led to the unambiguous characterization of several tryptic glycopeptides allowing the identification of the parent glycoproteins. Five of them, *i*.*e*. Rv0175, Rv0315, Rv0838 (LpqR), Rv1411c (LprG) and Rv3491, were then selected on the basis of the confidence of their glycopeptide identification (Supplementary Figure S1), their novelty with regards to previously characterized *O-*mannosylated *M*. *tuberculosis* proteins and their putative role in pathogenicity (Table 1). We reasoned that if *O-*mannosylation affects the function of a protein in a defined condition, then a knock-out mutation of the corresponding gene should also affect the phenotype in the same condition. Therefore, we individually disrupted each of the five target genes in the *M*. *tuberculosis* reference strain H37Rv and introduced a kanamycin resistant marker (*km*) together with an oligonucleotide specific tag by homologous recombination (Supplementary Figure S2). None of the mutants exhibited growth defects in standard ADC-enriched 7H9 liquid medium (Fig. 1A). However, the colony forming capacity of Δ*lprG* was impaired on 7H11 supplemented with OADC (Fig. 1B). This observation is similar to the strong growth defect on solid medium described for the H37Rv Δ*pmt* ^13^, although the phenotype was not as severe for the Δ*lprG* strain. None of the other mutants displayed growth alterations on solid medium (Supplementary Figure S3). Complementation of the Δ*lprG* mutant with the *lprG*-*Rv1410c* operon restored the capacity to form colonies on agar plates (Fig. 1B and Supplementary Figure S3).

**Table 1:** Selected *M*. *tuberculosis* mannoproteins with high confidence glycopeptidomics identification.

Peptide sequence	Experimental Mass (Da)	Calculated Mass (Da)	Delta (Da)	Hexose number	Peptide score	Protein Access #	Rv gene	Gene Name	Protein description	required for	
										*in-vitro* growth^d^	*in-vivo* growth^e^
D_100_C*VAA*T* ^a^QAPDAGAMSASMQK_119_^b^	2170,9	2008,9	162,1	1	121,2	O07419	Rv0175	mce1F	Mce associated membrane protein	−	+
P_39_AGP*T*PAPAAPAAA*T*GGLLFHDEFDGPAGSVPDPSK_74_	3544,7	3382,6	162,1	1	28,62	O07242	Rv0315^c^		β-1,3-glucanase precursor	−	+
*T*_47_ *TT*P*S*GPVPPV*S*EAAR_62_	1889,9	1565,8	324,1	2	55,3	O53850	Rv0838	lpqR	D-alanyl-D-alanine dipeptidase	−	nr
V_228_QV*T*KPPV*S*_236_	1115,6	953,6	162,1	1	24,94	P9WK45	Rv1411c^c^	lprG	Lipoarabinomannan carrier protein LprG	−	+
Q_164_PFSLQLIGPPPSPVQR_180_^b^	2022,1	1860,0	162,1	1	49,06	I6XHD1	Rv3491^c^		Predicted secreted protein	−	nr

*Carbamidomethyl cysteine; a: Underlined italic characters figure the potential glycosylation sites predicted by Net-O-Glyc 3.1 server; b: also reported by Smith *et al*., 2014; c: also detected by ref. 15; d: according^41, 42^; e: according^43, 44^; nr: not reported.

**Figure 2:**
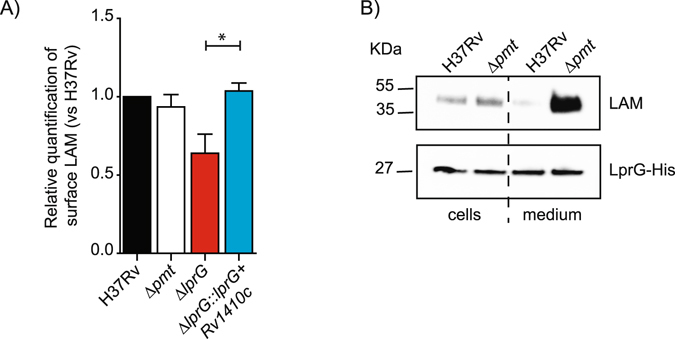
Impact of protein *O*-mannosylation on LAM exposition to the bacterial cell surface and release of the LprG/LAM complex. (**A**) FACS analyses of LAM staining on the surface of the various *M*. *tuberculosis* strains. The bar graph values correspond to the median fluorescence value after normalization to H37Rv. Results are representative of two independent experiments. *P < 0.05. (**B**) Western-blot analyses of LAM associated with LprG in WT *M*. *tuberculosis* or Δ*pmt* mutant. Similar amounts of LprG-His protein purified from the bacteria or culture medium were separated by SDS-PAGE. After transfer, the LAM or LprG-His molecules were revealed using the anti-LAM antibody CS-35 or an anti-His antibody. Blots are representative of at least three independent experiments.

One of the more striking feature of the *M*. *tuberculosis* Δ*pmt* mutant is its strong attenuation in cellular and animal infection models. We therefore evaluated the *in vivo* growth of the five mutants in BALB/c mice. We monitored the *in vivo* replication using qPCR in a competition experiment as described by Blumenthal *et al*.,^19^ to avoid any mis-interpretation associated with the altered capacity to form colonies on solid medium. Each mutant and the wild type strain was tagged using a specific oligonucleotide sequence. The tagged strains were grown independently in liquid medium and an inoculum was prepared by mixing similar quantities of each clone (Supplementary Figure S4). Mice were then infected intranasally using 6 × 10^2^ cfu of the mixed population (Supplementary Figure S4). At each time point, the bacterial load in lung and spleen was quantified by plating serial dilution of lung and spleen homogenates. As expected, we observed a five-log increase during the four-week-infection period (Figure S4). In parallel, the organ homogenates were used to grow a short-term culture used to extract total DNA and to evaluate the relative quantity of each strain by qPCR. The relative quantity of Δ*Rv0175* and Δ*lprG* was similar to that of the other mutants and wild type control in the inoculum, but their proportional representation decreased substantially during the infection time-course (Fig. 2C and Supplementary Figure S4). Conversely, the quantity of the other strains increased. These results show that the Δ*Rv0175* and Δ*lprG* mutants were attenuated in this competition experiment.

Based on these results, we focused our study on the LprG protein. Indeed, the phenotype of the H37Rv Δ*lprG* strain exhibited striking similarities with those of the Δ*pmt* mutant, suggesting that the latter may be due, at least in part, to lack of LprG mannosylation and subsequent alteration of LprG functions.

### Impact of O-mannosylation on LprG-associated localization of LAM

LprG belongs to the *M*. *tuberculosis* lipoprotein family and appears to act in concert with the efflux pump P55, encoded by the gene *Rv1410c*, which forms an operon together with *lprG* on the *M*. *tuberculosis* chromosome^20^. Several biological roles of LprG were recently described which provide tractable activities on which the impact of *O*-mannosylation can be evaluated. LprG together with P55 provides protection against toxic compounds such as ethidium bromide and malachite green^21, 22^. LprG is able to bind to various glycolipids such as lipoarabinomannan (LAM), lipomannan and phosphatidylinositol mannoside (three groups of molecules which share a similar core structure)^23^ and triglycerides^24^. LprG mutants exhibit impaired surface exposition of LAM^25, 26^, suggesting that the biological role of this lipoprotein is to transfer LAM from the plasma membrane to the bacterial cell surface.

We examined whether the lack of *O*-mannosylation alters one or more functions of LprG. We first evaluated the presence of LAM at the surface of the WT H37Rv strain and the Δ*pmt* and Δ*lprG* isogenic mutants using FACS (Fig. 2A). As expected, disruption of *lprG* decreased the surface display of LAM, a phenotype reversed by complementation with the *lprG-Rv1410c* operon. However, the Δ*pmt* strain exhibited a level of LAM exposition similar to that of the WT strain, suggesting that the lack of LprG glycosylation does not prevent the translocation of LAM. We further explored the impact of glycosylation on LprG function by testing whether co-purification of LAM with LprG was affected in a mycobacterial strain deficient for *O*-mannosylation. We expressed LprG fused to a Hexahistidine (His)-tag in the WT H37Rv or the Δ*pmt* isogenic mutant. We then affinity purified the LprG-His protein from either the culture filtrate or the bacterial cells and measured the amount of LAM co-purified with LprG-His by Western blot using an anti-LAM antibody. Similar amounts of LAM were associated with the LprG-His protein purified from the pellet of the Δ*pmt* mutant and the WT strain (Fig. 2B). In sharp contrast, the amount of LAM associated with LprG-His recovered from the culture filtrates was much higher for the Δ*pmt* mutant than for the WT strain. This suggests that the *O*-mannosylation defect favors the release of the LAM associated with LprG in the bacterial culture filtrate. We tested whether this effect was restricted to *M*. *tuberculosis* by expressing the LprG-His protein in *M*. *smegmatis* mutated for the *MSMEG*_*5447* (the ortholog of *Rv1002c*) gene (*MSMEG-5447::res* or Δ*pmt* mutant) or the isogenic strain complemented with *Rv1002c* from *M*. *tuberculosis*. Similar amounts of LAM were co-purified with LprG-His from the pellets of the *M*. *smegmatis* Δ*pmt* mutant and the *Rv1002c-*complemented strain, whereas much higher LAM were associated with the protein purified from the Δ*pmt* mutant culture supernatant, as observed in the *M*. *tuberculosis* background (Supplementary Figure S5).

Altogether, these results suggest that the lack of *O*-mannosylation increased the amount of LAM released in the supernatant in association with LprG.

### Identification of the LprG glycosylation site

**Figure 3:**
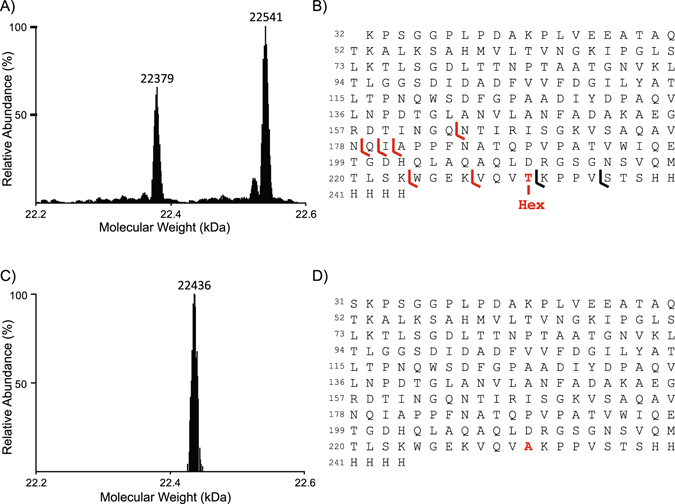
MS analyses of LprG and LprG T231A glycosylation. (**A**) Deconvoluted ESI-HRMS spectrum of the purified recombinant LprG-His expressed in *M*. *tuberculosis*. (**B**) Peptide sequence of the molecular species observed in A reporting the fragment ions detected in the top-down electron transfer dissociation spectrum of the fragmentation of the 22,541 Da molecular mass ion precursor allowing the localization of the unique hexose on the T231. (**C**) Deconvoluted ESI-HRMS spectrum of purified recombinant LprG-His T231A expressed in *M*. *smegmatis* demonstrating the absence of glycosylation of the mutated protein. (**D**) Peptide sequence of the molecular species observed in C.

We tested whether the lack of LprG *O*-mannosylation was responsible for the higher association of LAM with LprG observed with the Δ*pmt* mutant, by targeting specifically the LprG glycosylation. We first further characterized the LprG mannosylation status and precisely determined the glycosylation site(s) by high resolution mass spectrometry (HR-MS) and top-down fragmentation experiments of the intact protein. Deconvolution of the ESI-HRMS spectrum of the purified recombinant LprG-His expressed in *M*. *tuberculosis* revealed two molecular species of molecular mass of 22,379 Da and 22,541 Da (Fig. 3A and B). The 162 Da difference in mass between these two peaks is consistent with the mass of an hexose moiety, suggesting that these two molecular species correspond to the unmodified and monoglycosylated proteoforms of a truncated form of LprG (Fig. 3A and B) missing the N-terminal pentapeptide (expected average mass of 22,379 Da and 22,541 Da). Top-down fragmentation of the intact protein precursors supported these assignments and further allowed the identification of T231 as the unique glycosylation site of the glycoform 22,541 Da protein (Figs 3B and S6). We produced a *lprG* A691G point mutant (lprG*), encoding a LprG protein containing a T231A mutation, fused with a His-tagged coding sequence, to evaluate the impact of the mannosylation on the activities of the protein. This construct was expressed in the *M*. *smegmatis* Δ*pmt* mutant complemented with *Rv1002c* and the LprG-His T231A protein was purified and analyzed by MS in a same way. As expected, we detected a unique molecular species with a molecular mass of 22,436 Da (Fig. 3C and D) consistent with a mutated T231A form of LprG, confirming that the mutated protein was no longer *O*-mannosylated. However, although the molecular mass of this molecular species is consistent with the T231A mutation, it is noteworthy that it corresponds to a slightly longer truncated form of LprG starting from the upstream N terminal serine residue (Fig. 3C and D). This alternative sequence truncation was confirmed by top-down MS experiments and suggests that the absence of glycosylation may affect the proteolytic cleavage of the protein N terminus as previously reported^5^.

### Impact of LprG O-mannosylation on mycobacterial growth and association with LAM

**Figure 4:**
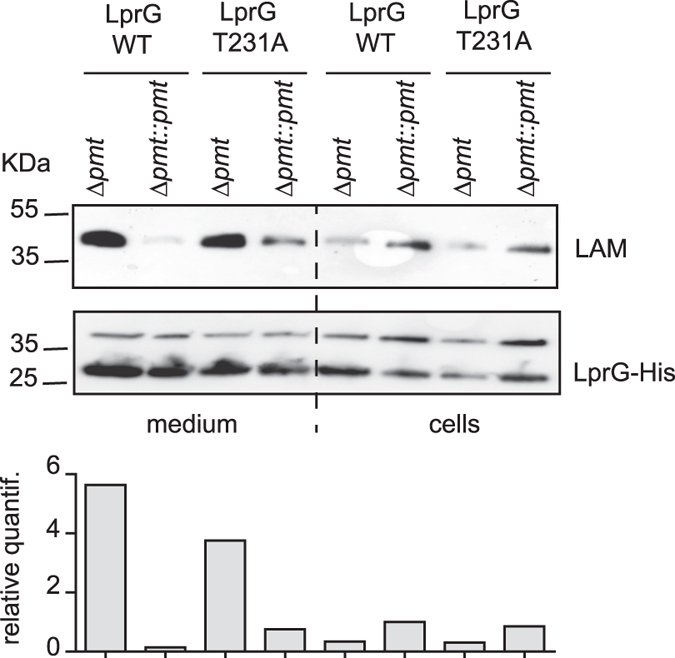
Impact of LprG *O*-mannosylation on the release of the LprG/LAM complex in *M*. *smegmatis*. Similar amounts of LprG-His or LprG-His T231A purified from either culture medium or bacterial cells were separated by SDS-PAGE, transferred onto membranes and revealed using anti-LAM or anti-His antibodies. The genetic backgrounds in which the protein was produced are indicated. The upper band revealed by the anti-His antibody is a non-specific cross-reacting protein. The signal intensity for LAM was quantified and plotted in the graph below the Western blot image. This blot is representative of three independent experiments.

We next addressed whether the lack of LprG glycosylation directly affects its functions. We generated a complementation plasmid carrying the lprG* mutated gene in an operon with *Rv1410c* and transferred this construct into the H37Rv Δ*lprG* mutant. We verified whether the production of LprG T231A complements the Δ*lprG* mutation. We first evaluated their colony-forming capacity on 7H11 OADC solid medium and found no difference between the strains producing the wild type LprG or the LprG T231A mutated protein, indicating that the LprG T231A is functional (Supplementary Figure S7). We next tested whether the T231A mutation affects the capacity of LprG to co-purify with LAM. The LprG-His and LprG-His T231A proteins were purified either from the *M*. *smegmatis* Δ*pmt* mutant or from the isogenic strain complemented with *Rv1002c*. Western-blot analyses revealed that similar amount of LAM co-purified with LprG-His and LprG-His T231A recovered from the same *M*. *smegmatis* background. However, the LAM signal was much higher when the proteins were purified from the supernatant of the Δ*pmt* mutant than complemented *M*. *smegmatis* (Fig. 4). In contrast, the amount of LAM in the cell extracts was slightly higher for the complemented strain than for the Δ*pmt* mutant suggesting that the LAM/LprG complex is more efficiently retained in the cells when protein-*O*-mannosylation is preserved (Fig. 4).

Altogether these results establish that the enhanced capacity of LAM to co-purify with secreted LprG when produced in *M*. *smegmatis* Δ*pmt* mutants is not due to LprG mannosylation but rather to an indirect effect.

### Impact of protein O-mannosylation on steady state level of LAM

**Figure 5:**
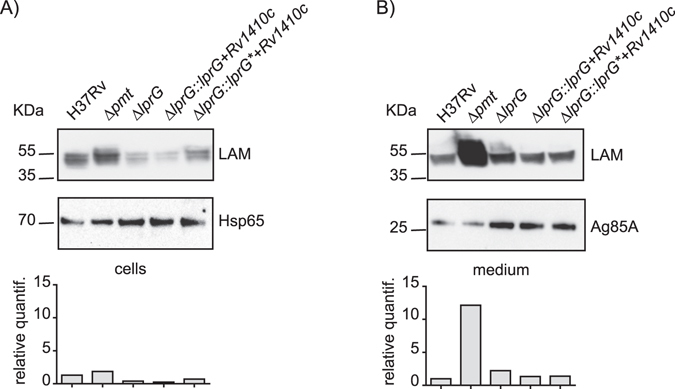
Impact of protein *O*-mannosylation on LAM production. Crude extracts obtained from the culture medium (**B**) or bacterial cells (**A**) were separated by SDS-PAGE, transferred onto membranes, and analyzed using antibodies raised against LAM and either hsp65 (for cellular extracts) or Ag85A (for medium supernatants). The signal intensity for LAM was quantified and plotted in the graph below the western blot image. This blot is representative of two independent experiments.

The higher amount of LAM found associated with LprG in the supernatant of the Δ*pmt* mutants might be due to a higher steady state level of these compounds. We evaluated the total amount of LAM found in the *M*. *tuberculosis* WT, Δ*pmt* and Δ*lprG* mutants and complemented strains. We prepared crude extracts from bacterial pellets or culture supernatants, separated the compounds by SDS-PAGE, and then performed Western blot analysis using either anti-LAM, or anti-hsp65 and anti-Ag85A antibodies as controls for the cell-associated or extracellular proteins, respectively (Fig. 5). As expected, the bacterial pellets contained similar amounts of LAM, but the culture supernatant of the Δ*pmt* mutant exhibited approximately 10 fold more LAM than that of the WT cells. This phenotype was reverted by complementation with *pmt* from either *M*. *tuberculosis* or *M*. *smegmatis* (data not shown). In contrast, the amount of LAM was similar in both fractions for the other mutant and complemented strains, including the strain expressing the LprG-His T231A. This finding was not restricted to *M*. *tuberculosis* because similar results were obtained for crude extracts from *M*. *smegmatis* WT and the Δ*pmt* mutant (data not shown). These results demonstrate that the production of LAM and its release into the culture medium is enhanced in the Δ*pmt* mutants, independently of the LprG mannosylation.

### Protein O-mannosylation and induction of inflammatory response

**Figure 6:**
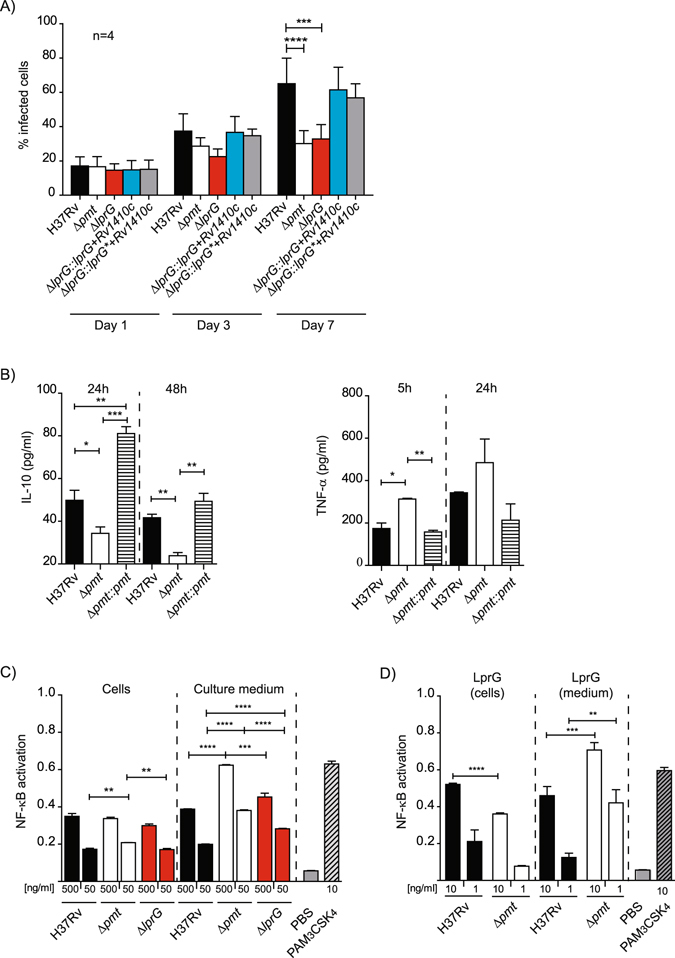
Protein *O*-mannosylation affects the inflammatory response of infected cells. (**A**) Growth of WT, mutants and complemented strains in hMDM. hMDM were infected at an MOI 1 for 1 h with the indicated strains. Data are presented as the mean +/− SD of four independent experiments performed in duplicate. (**B**) Production of TNF-α and IL-10 by MHS cells infected by WT, Δpmt and complemented strains. MHS cells were infected at MOI 1:1 for 1 h and TNF-α and IL-10 were evaluated after 5 h or 24 h and 24 or 48 h respectively. Data are representative of two independent experiments performed in triplicate. (**C**) NK-κB activation in HEK-TLR2 cells incubated with crude extracts from WT and Δ*pmt M*. *tuberculosis* strains. Activation of NF-κB, evaluated using SEAP activity, following incubation with 500 ng/ml or 50 ng/ml of crude extracts recovered from either bacterial cells or culture medium from the indicated *M*. *tuberculosis* strains. PBS 1× or PAM_3_CSK_4_ (10 ng/ml) were used as negative and positive controls respectively. Data are presented as the mean +/− SD of three independent experiments. (**D**) NF-κB activation in HEK-TLR2 cells incubated with LprG/LAM complex purified from bacterial cells or culture medium of WT or Δ*pmt M*. *tuberculosis* strains. HEK-TLR2 cells were incubated with 10 ng/ml or 1 ng/ml of LprG-His and co-purified LAM. SEAP activity was quantified after 24 h. PBS 1× or PAM_3_CSK_4_ (10 ng/ml) were used as negative and positive controls respectively. Data are presented as the mean +/− SD of at least five independent experiments. *P < 0.05; **P < 0.01; ***P < 0.001; ****P < 0.0001.

LprG plays a key role in the cross-talk between *M*. *tuberculosis* and the host by interacting with the host receptor, TLR2, and by modulating the inflammatory response^23, 27^. This TLR2 agonist activity is strongly enhanced by LAM association with LprG^23^. The higher release of LAM/LprG complex may be associated with modification of the interaction of *M*. *tuberculosis* Δ*pmt* mutant with macrophages. We first infected human monocyte-derived macrophages (hMDM) at an MOI of 1:1 for 1 h with the Δ*pmt* or Δ*lprG* mutants, the WT control or the complemented strains and monitored the percentage of infected cells and the number of bacteria per cell for seven days. For this purpose, the strains were transformed with a plasmid expressing the fluorescent protein GFP. As we previously observed, the Δ*pmt* mutant showed impaired invasion and growth in hMDM^13^. The *ΔlprG* mutant exhibited a similar defect of invasion capacity (lower percentage of infected cells and lower number of bacteria/cell) after seven days of infection. Complementation with either the WT *lprG* or mutated *lprG* A691G in operon with *Rv1410c* restored full virulence in this cellular model (Fig. 6A and Supplementary Figure S8). These results demonstrate that the lack of LprG *O*-mannosylation was not responsible for the attenuation observed with either the Δ*lprG* or Δ*pmt* mutant.

We next evaluated whether the inflammatory response of infected cells was affected by the Δ*pmt* mutation. We used cell lines to limit the variability associated with primary cells. We infected human (THP-1) or mice macrophages (MHS) at MOI 1:1 for 1 h with either the wild type H37Rv, the Δ*pmt* mutant, and the *Rv1002c*-complemented strain and assayed inflammatory (TNF-α) or anti-inflammatory (IL-10) cytokines at 5, 24 and 48 h post-infection. We detected a significantly greater TNF-α release by MHS cells after infection with the Δ*pmt* mutant than with the WT or complemented strains and a similar trend was observed in THP-1 cells (Fig. 6B and Supplementary Figure S8). In contrast, the secretion of the anti-inflammatory cytokine IL-10 decreased markedly in both cell types. We next addressed whether this higher inflammatory response may be due to greater stimulation of TLR2 using a HEK293 cell line stably transfected with human TLR2 and CD14 genes and an NF-κB-inducible reporter gene. We prepared crude extracts from the bacterial pellets and culture supernatants of Δ*pmt*, Δ*lprG* mutants, and controls and incubated them with HEK-TLR2 cells. The culture supernatants from the Δ*pmt* mutant activated NF-κB more than the similar extract from the WT strain (1.6-fold increase, p < 0.001) (Fig. 6C). In contrast, similar TLR2 activation was observed for the Δ*lprG* mutant and strains producing either the WT LprG or the non mannosylated LprG T231A (data not shown). The difference observed for the Δ*pmt* sample was restricted to the culture supernatant because the crude extracts obtained from the various bacterial pellets gave similar NF-κB activation. These results are consistent with the higher secretion of the LprG/LAM complex associated with the Δ*pmt* mutation.

We next tested the LprG-His protein purified from the culture medium or bacterial pellets from the WT *M*. *tuberculosis* or the Δ*pmt* mutant for their TLR2 agonist activity (Fig. 6D). NF-κB activation was slightly higher for the proteins purified from the WT bacterial pellets than the Δ*pmt* cell*s* when equal amounts of LprG-His were incubated with HEK-TLR2 cells. In contrast, we measured much higher NF-κB activation with LprG-His purified from the supernatant of the H37Rv Δ*pmt* mutant than from the supernatant of the WT strain (1.5-fold increase, p < 0.001 for 10ng/ml purified proteins and 3.4-fold increase, p < 0.005 for 1 ng/ml). We obtained similar results with LprG-His purified from either the bacterial pellet or culture supernatant from the *M*. *smegmatis* Δ*pmt* mutant or the isogenic strain complemented with *Rv1002c* (Supplementary Figure S9). The T231A mutation, and as a consequence the glycosylation status of LprG, did not affect the induction of NF-κB (data not shown).

Finally, we confirmed that LprG/LAM signals primarily through TLR2 using bone marrow-derived macrophages (BMDM) from WT, TLR2 −/−, or MyD88−/− mice. Incubation of WT BMDM with the LprG/LAM complex induced the production of TNF-α. Consistent with results obtained with HEK-TLR2 cells, we measured higher TNF-α release with LprG/LAM purified from culture medium from the *M*. *smegmatis* Δ*pmt* than from the *Rv1002c*-complemented strain in WT macrophages and the opposite for samples obtained from the bacterial pellets. The TNF-α release was abrogated in TLR2 −/− BMDM as in macrophages deficient for MyD88, the common adaptor protein for most TLR (Supplementary Figure S10).

Altogether, these results clearly establish that the lack of protein glycosylation is associated with a greater induction of the TLR2-mediated inflammatory response during host-cell infection. This effect is explained, at least in part, by the higher release of the LAM/LprG complex.

## Discussion

The molecular mechanisms explaining the various phenotypes associated with the lack of protein *O*-mannosylation in mycobacteria are unknown. In *M*. *tuberculosis*, these phenotypes include a marked deficiency to form colony and severe attenuation in mouse and cellular models of infection^13^. Here, we demonstrated that the *M*. *tuberculosis* Δ*pmt* mutant induces a stronger inflammatory response by infected phagocytes than the WT strain. This pro-inflammatory phenotype may be explained by higher secretion of the LAM/LprG complex which stimulates the TLR2 signaling pathway leading to NF-κB activation. Although LprG is *O*-mannosylated, this modification does not influence release of the LAM/LprG complex. Rather, disruption of mycobacterial *pmt* leads to a higher steady-state level of LAM and release in the supernatant of mycobacterial cultures. The additional LAM in the medium, at least in part bound to LprG, is likely the molecular basis underlying the greater induction of the inflammatory response through potentiation of the TLR2 agonist activity of LprG.

This conclusion is supported by several lines of evidence. First the greater inflammatory response induced in human and mouse macrophages by the *M*. *tuberculosis* Δ*pmt* mutant was reproduced in a simpler system using either non phagocytic HEK-TLR2 cells or BMDM from WT or TLR2 −/− mice and either the mycobacterial culture supernatant or the purified LprG/lipoglycan complex. Second, the T231A point mutation abolished LprG *O-*mannosylation but had no effect on NF-κB activation and accumulation of LprG/LAM complex. Finally, although the LprG lipoprotein was found to exhibit TLR2 agonist activity^23, 27^, association with mycobacterial lipoglycans (including LAM) strongly enhanced this activity^23^. However, further studies are required to determine whether this stronger induction of the TLR2 mediated response accounts for the attenuation observed with the *M*. *tuberculosis* Δ*pmt* mutant in the mouse model. In the last decade, TLR2 has emerged as a major host receptor for host/pathogen cross talk and immune response against *M*. *tuberculosis* (for review^28, 29^). TLR2 signaling mediates the control of *M*. *tuberculosis* through a wide range of host immune responses including macrophage activation, pro-inflammatory cytokine release, Th1 response and apoptosis^28^. Accordingly, TLR2-deficient mice exhibit higher susceptibility to *M*. *tuberculosis* infection than WT mice^18, 30^. However, *M*. *tuberculosis* may also use TLR2 signaling to escape the host immune response. TLR2 agonists from *M*. *tuberculosis* were found to suppress dendritic cell activation, type I interferon induction and antigen processing and presentation^31, 32^. The fine tuning of the inflammatory response, in particular through TLR2 signaling, is thought to be a strategy developed by the tuberculosis bacillus to achieve the various steps of its infectious cycle, from survival of the initial encounter with the host, to persistence in granulomas and finally to the induction of the central necrosis and rupture of the granuloma favoring bacterial release in the airways and transmission^33^. Perturbation of this fine tuning may be deleterious to the pathogen through increased production of toxic compounds, but also to the host through uncontrolled inflammation^34, 35^.

A surprising observation from this study was that several phenotypes associated with the *Δpmt* mutation are also found for the *ΔlprG* mutant, including the defect in colony formation on solid media and a similar level of attenuation in hMDM. However, our analyses with the strain producing LprG T231A, which is no longer glycosylable by Pmt, strongly argues against a direct role of LprG glycosylation in the corresponding phenotype. It is possible that the activity of a protein acting in the same pathway as LprG is strongly influenced by *O-*mannosylation. An obvious candidate is the efflux pump encoded by the gene *Rv1410c* in *M*. *tuberculosis* H37Rv. This gene forms an operon together with *lprG* and most of the phenotypes described for the *ΔlprG* mutant were only complemented upon expression of both *Rv1410c* and *lprG* (this study^21, 22^). We examined our mass spectrometry data in depth but found no evidence that the *Rv1410c*-encoded protein is *O-*mannosylated (data not shown). The impact of glycosylation on LprG activity thus remains an open question.

Another unexpected result from this study was the increased LAM production in the Δ*pmt* mutants. This finding is not restricted to *M*. *tuberculosis* H37Rv because we observed the same for the *M*. *smegmatis* Δ*pmt* mutant. Most of the additional lypoglycans were found in the culture supernatant. As discussed before, this enhanced secretion has a major impact on the inflammatory response of the host cell but the molecular basis for such abnormal production and secretion remains unclear. None of the enzymes known to be involved in the LAM biosynthetic pathway were identified among the *O*-mannosylated proteins (data not shown). One possible explanation may reside in the mannose flux. Indeed, Pmt is thought to transfer mannose residues from the same decaprenyl-phospho-mannose donor used for the elongation of PIM during the formation of LM and LAM^36^. The lack of Pmt may increase the amount of decaprenyl-phospho-mannose donor available for these other pathways. Another possibility is that genes of the LAM biosynthesis pathways are upregulated in the Δ*pmt* strains. This is still speculative and additional work would be required to establish this point. However, such side effect was previously observed in mycobacteria for other mutants. For example, mutations that abolish the synthesis or transport of glycopeptidolipid in the opportunistic pathogen *Mycobacterium abscessus* enhance the production of lipoproteins which, through their TLR2 agonist activity, stimulate an inflammatory response during host cell infection^37^.

In conclusion, protein *O*-mannosylation appears to modulate the production and secretion of inflammatory molecules such as LAM, but the underlying molecular mechanism remains to be established. The exposure of these lypoglycans, which act as strong TLR2 ligands, especially when associated with the lipoprotein LprG leads to modulation of the innate immune response, providing a possible explanation for the strong attenuation of the Δ*pmt M*. *tuberculosis* strain in cellular and animal infection models.

## Materials and Methods

### Bacterial growth conditions

*M*. *smegmatis* mc^2^155 wild-type (WT) and *M*. *smegmatis* derivatives were cultured in Luria Bertani (LB) broth containing 0.05% Tween-80 or LB agar at 37 °C. *M*. *tuberculosis* WT and *M*. *tuberculosis* mutants were grown at 37 °C in liquid Middlebrook 7H9 broth (Difco) containing ADC (Becton Dickinson) and supplemented with 0.05% Tween-80 or in solid Middlebrook 7H11 broth supplemented with OADC (ADC with 0.005% oleic acid) (Becton Dickinson). Albumin free culture conditions for the *M*. *tuberculosis* strains were performed in 7H9 broth supplemented with dextrose (1 g/L) and catalase (1.5 mg/L). For analysis of LAM, bacteria were grown without Tween-80. When necessary, media were supplemented with Hygromycin (Hyg) and Kanamycin (Km) at 50 µg/ml and 40 µg/ml respectively. Liquid cultures were grown to logarithmic or stationary phases depending of the needs.

*E*. *coli* DH5α was used for cloning experiments. Strains were grown at 37 °C in LB broth or LB agar. When necessary, Hyg (100 µg/ml) and Km (40 µg/ml) were added for selection of the appropriate strains.

In order to characterize the growth rate of *M*. *tuberculosis* WT and *M*. *tuberculosis* mutants, growth curves were monitored in 50 mL of 7H9 ADC 0.05% Tween-80. *M*. *tuberculosis* strains were grown until log-phase and then inoculated at 10^6^ cfu/mL. OD 600 nm was measured at different time points for one month. The capacity of the different mutants to grow on solid media was evaluated on 7H11 OADC. *M*. *tuberculosis* cultures were grown until exponential phase, OD was measured and bacterial concentration was adjusted to 10^6^ cfu/mL. Then, 10 μL of serial dilutions were plated. Morphology and size of spots were evaluated at 2–4 weeks of growth.

Every experiment involving WT or recombinant *M*. *tuberculosis* strains were performed in BSL3 laboratories until the samples were inactivated. Experiments with *E*. *coli* or *M*. *smegmatis* were realized in BSL1 laboratories.

### Construction of *M*. *smegmatis* and *M*. *tuberculosis* mutants and complementation

*M*. *tuberculosis* knockout (KO) strains were constructed by homologous recombination using the recombineering system^38^. First, the allelic exchange substrate (AES) for each gene of interest: *Rv0175c*, *Rv0315*, *Rv0838*, *Rv1411c* and *Rv3491* were produced. Primers were designed with restriction sites to amplify upstream and downstream regions of the targeted genes (Supplementary Table S1) and PCR fragments were ligated to a Km resistance cassette. Then, *M*. *tuberculosis* H37Rv carrying a pJV53 derivative containing the *hyg* resistance gene was grown to mid-log phase, induced with acetamide and competent cells were prepared. This strain was transformed with each AES. Km-resistant colonies were obtained and replacement of the targeted gene by the AES was verified by PCR analysis (Supplementary Figure S1, Supplementary Table S1). As a result, *M*. *tuberculosis* ΔRv0175, ΔRv0315, ΔRv0838 (Δ*lpqR*), ΔRv1411c (Δ*lprG*) and ΔRv3491 mutants were obtained.

*M*. *tuberculosis* Δ*lprG* was complemented with *Rv1410c*, the *lprG* gene (*Rv1411c*) or both genes. Briefly, PCR products were amplified from *M*. *tuberculosis* H37Rv total DNA using primers indicated in Table S1 and inserted between *PvuII* and *HindIII* restriction sites of the integrative plasmid pMV361H^39^. In this construct, the gene expression is under the control of its own promoter.

*M*. *tuberculosis* H37Rv, Δ*lprG* and Δ*pmt gfp* strains were made by insertion of the pMV361H GFP integrative plasmid. The various complemented strains expressing GFP were made using pMV361H GFP integrative plasmid carrying the PCR fragment of interest inserted between *PvuII* and *HindIII* sites (Supplementary Table S2).

Integrative plasmid pMV361H *Rv1410c-lprG* A691G *gfp* was constructed using the pMV361H *Rv1410c-lprG gfp* as template. Briefly, the whole plasmid was amplified using CGAGAAGGTCCAGGTCGCGAAGCCCCCGGTGAGCTGATC and GATCAGCTCACCGGGGGCTTCGCGACCTGGACCTTCTCG primers. Then, the PCR product was transformed in *M*. *tuberculosis* Δ*lprG* to obtain *M*. *tuberculosis* Δ*lprG*:: *Rv1410*c-*lprG* A691G *gfp* strain.

*M*. *smegmatis_5447::res-Ωkm-res* was complemented with pWM158 integrative plasmid which contains the *Rv1002c* gene from *M*. *tuberculosis* ^13^.

### Mouse Infections and qPCR analysis

Female BALB/c mice (7–8 week old) were purchased from the Centre d’Elevage Janvier and housed in the Institut de Pharmacologie et de Biologie Structurale ASB3 animal facilities. The *M*. *tuberculosis* WT and mutant strains were grown independently in standard liquid medium and an inoculum was prepared by mixing similar quantity of each clones. Mice, five per group, were infected intranasally with 40 μL PBS containing 6 × 10^2^ cfu of the mixed population. At 1, 14 and 28 days post-infection, 5 mice were euthanized and lungs and spleens were homogenized in 5 mL of PBS 0.05% Tween-80. The course of the global infection was followed by plating serial dilutions of 100 μL of lung and spleen homogenates on 7H11 OADC solid medium. The remains of organ homogenates were centifugated at 800 rpm to remove clumps. The supernatants were then centifugated at 14000 rpm and bacilli were resuspended in 5 mL of 7H9 ADC Km. After one week of culture, mycobacterial DNA were extracted using DNeasy Blood and tissue extraction kit (Qiagen) according to the recommendations of the manufacturer.

Quantitative PCR analysis was performed in 25 μL reactions using the suitable set of primers (Supplementary Table S3), 5 μL of DNA as template and the maxima syber green qPCR master mix (thermo scientific). Reactions were performed on the Applied Biosystems 7500. The amplification program for qPCR was: 10 min at 95 °C, followed by 40 cycles each consisting of 95 °C for 15 sec, 60 °C for 30 sec and 72 °C for 30 sec. Two PCR reactions were used for each mutant. The expression of the common part was used as internal control (qKmFint and qKmRint primers). Specific values were first normalized with the common region and relative quantification was then calculated.

### Cell Culture and infections

Human monocyte-derived macrophages (hMDMs) were prepared as previously described^40^. Briefly, human blood samples, obtained from the Etablissement Français du Sang of Toulouse (France), were collected from anonymous nontuberculous donors. Peripheral blood monocytes were cultured to differentiation for 7 days on glass coverslips in 24-well tissue culture plates (5.10^5^ cells/well) containing RPMI 1640 (Gibco, Cergy Pontoise, France) supplemented with 2 mM glutamine (Gibco) and 7% of heat-inactivated human AB serum. Before infections, hMDMs were washed twice with fresh RPMI medium. Bone marrow-derived macrophages from C57Bl6 (WT), TLR2 −/−, or MyD88−/− mice were differentiated in DMEM (Invitrogen) supplemented with 10% FCS (Fisher Scientific), 20% Macrophage Colony Stimulating Factor (M-CSF from L929 cell supernatant) and 10 mM HEPES (Invitrogen). The day before stimulation with LprG/LAM complex, macrophages were seeded into 96-well plates at a density of 5.10^4^ cells/well.

Mycobacterial infections using hMDMs were performed as previously described^40^. Briefly, macrophages were infected with exponentially growing GFP-expressing mycobacteria at MOI 1 :1 in RPMI medium. Infection was allowed to proceed at 37 °C in 5% CO_2_ for 1 h, and the extracellular bacteria were removed by three successive washes with 1 mL fresh medium. At the end of the infection period, the cells were collected at 0, 3 and 7 days post-infection and fixed for 1 h at RT with 4% PFA in PBS supplemented with 15 mM sucrose, aldehyde groups were neutralized with 50 mM NH_4_Cl. Extracellular mycobacteria were labelled with rabbit anti-mycobacteria Ab, which was detected by a Rhodamine Red-conjugated goat anti-rabbit secondary Ab. Preparations were visualized under a Leica DMLA fluorescence microscope to count and calculate (i) the number of bacteria per macrophages and (ii) the number of macrophages containing at least one particle (percent internalization or phagocytosis). Data are expressed as relative values, calculated as the percentage of internalization or phagocytosis divided by control values. For each set of conditions, triplicate experiments were performed and at least 50 cells per slide were counted.

### Quantification of NF-κB activity and cytokine secretion (TNF-α and IL-10)

The level of NF-κB activation was studied using HEK-Blue^TM^ hTLR2 reporter cell lines (InvivoGen). This cell line is stably transfected with a reporter plasmid expressing a secreted embryonic alkaline phosphatase (SEAP) gene under the control of a NF-κB-dependent promoter. In all cases, cells were used in 96-well plates according to manufacturer instructions. Cells were treated with the purified proteins at the specified concentrations. Unstimulated cells were used as negative controls, whereas Pam3CSk4 10 ng/ml was used as positive control. After 16 h of incubation, QUANTI-Blue^TM^ detection medium (InvivoGen) was used following manufacturer specifications and SEAP activity, corresponding to NF-κB activation, was determined by reading the absorbance at 630 nm. For infection experiments, HEK-Blue^TM^ hTLR2 cells were infected with corresponding *M*. *tuberculosis* strains in 96-well plates at MOI 1:1 for 24 h. At the tested infection time, supernatants were harvested, filtered, and NF-κB activity was subsequently evaluated as described before.

To assess TNF-α or IL-10 secretion levels, THP-1 or MHS cells were infected at MOI 1:1 for 1 h and cytokines were evaluated after 5 h and 24 h for TNF-α and 24 and 48 h for IL-10. Cytokine secretions were quantified in supernatants of the THP-1 or MHS cells using the BD OptEIA^TM^ human TNF ELISA Set and BD OptEIA^TM^ human IL-10 ELISA set or the PeproTech murine TNF-α and IL-10 ABTS ELISA development kits respectively, according to the recommendations of the manufacturer. For BMDM, cells were incubated for 24 h with 100 ng/ml LprG/LAM complexes purified from bacterial cells or culture medium of *M*. *smegmatis* Δ*pmt* or *M*. *smegmatis* Δ*pmt* complemented with *pmt* from *M*. *tuberculosis*. Unstimulated cells were used as negative controls (PBS 1X), whereas Pam3CSK4 (100 ng/ml) and LPS (100 ng/ml) was used as positive control. TNF-α secretion was quantified in supernatants of the BMDM using the mouse TNF-α ELISA Set kit (eBioscience), according to the recommendations of the manufacturer. Data are expressed as picograms of cytokine per milliter of supernatant.

### Cloning, expression and purification of His-tagged proteins

Cloning, expression and purification of LprG and LprG T231A His-tagged proteins were performed as previously described with minor modification^23^. The *lprG* gene was amplified from *M*. *tuberculosis* H37Rv reference strain by PCR using the 5′-TTTTTTCATATGCGGACCCCCAGACGCCACTG-3′ and 5′-TTTTTTACTAGTGCTCACCGGGGGCTTCG-3′ primers. The *lprG* A691G (lprG*) was amplified from the complementation plasmid using the same primers (underlined the *NdeI* and *SpeI* restriction enzyme sites). Both DNA fragments were inserted between the *NdeI* and *SpeI* sites of pWM218 mycobacterial replicative plasmid^13^ to obtain pLprG-His and pLprG T231A-His plasmids. The *lprG* gene expression is under the control of the pBlaF* promoter.

LprG and LprG T231A were expressed either in *M*. *tuberculosis* or in *M*. *smegmatis*. Cultures were centrifuged and pellet and medium were separated. Supernatants were filtered twice and concentrated to 2 mL by ultrafiltration with Vivaspin 10 kDa system (Sartorius). Cells were resuspended in 2 mL of PBS 1x pH 7.4 and disrupted. Insoluble material was removed from the lysate by centrifugation at 12000 rpm for 10 min at 4 °C and supernatant was incubated directly with Ni^2+^ beads (Qiagen) for 2–4 h at 4 °C in continuous agitation. The mix was transferred to polypropylene columns, washed with 5 volumes of wash buffer (PBS, 20 mM imidazole, pH7.4), and bound proteins were dissociated with aliquots of 100 μl of elution buffer (PBS, Imidazole from 100 to 500 mM). After purification, proteins were dialyzed 3 times in PBS pH 7.4. Protein purity was verified by SDS-PAGE with blue-Coomassie stain and Western-blot using anti-His antibody.

### LAM detection in crude extracts

*M*. *tuberculosis H37Rv*, *Δpmt*, *ΔlprG*, *ΔlprG:: lprG-Rv1410c* and ΔlprG:: lprG* -Rv1410c were grown in 7H9 supplemented with ADC during one month. Cultures were centrifuged and pellets and medium were separated. Supernatants were filtered and concentrated to 2 mL by ultrafiltration with Vivaspin 10 kDa system (Sartorius). Bacterial pellets were resuspended in 2 mL of PBS 1x pH7.4 and disrupted. Insoluble material was removed from the lysate by centrifugation at 12000 rpm for 10 min at 4 °C. The total amount of LAM was analyzed by Western blot using the CS-35 anti-LAM antibody. Anti-hsp65 or anti-Ag85A antibodies were used as controls.

### Western blot analysis

Western blot analysis was carried out using standard procedures. Equivalent quantities of protein extracts or purified proteins were separated by electrophoresis through a 4–20% precast protein gel (BioRad). Proteins were transferred to PVDF membranes for 50–60 min. After 1 h blocking with 5% milk in PBS supplemented with 0.05% Tween-20 (PBST), proteins were analyzed using specific antibodies: mouse monoclonal anti-LAM (CS-35, BEI Resources, 1:50, overnight at 4 °C), mouse monoclonal anti-His6 (Affymetrix, 1:3000, overnight at 4 °C), mouse monoclonal anti-Ag85 (Santa Cruz Biotechnology, 1 :1000, overnight at 4 °C), rabbit polyclonal anti-Hsp65 (gift from M. Daffé, 1:2000000, overnight at 4 °C) goat anti-mouse (BioRad, 1 :5000, 1 h RT) or goat anti-rabbit (Sigma-Aldrich, 1:20000, 1 h RT) secondary antibodies conjugated with horseradish peroxidase (HRP).

### Surface LAM staining

Ten days cultures were centrifuged at 800 rpm for 10 min to remove clumps. Bacilli were washed with PBS and fixed in 4% PFA. After centrifugation, pellets were washed with PBS and incubated at RT in 5% BSA for 1 h. Bacilli were sedimented and suspended in 3% BSA with mouse anti-LAM CS-35 at 1/150 and incubated overnight at 4 °C. Bacilli were washed with PBS-0.05% Tween-20 and resuspended in PBS with goat anti-mouse IgG CMA-Rho red at 1/1000 for 2 h at RT. Bacilli were washed and analyzed by flow-cytometry. Relative quantification of LAM was calculated using H37Rv as reference level.

### High-Resolution Mass Spectrometry

LC-ESI-MS and MS² analyses of intact recombinant LprG-His and LprG-His T231A were performed on a nanoRS UHPLC system (Dionex) coupled to an ETD-enabled LTQ-Orbitrap Velos mass spectrometer (Thermo Fisher Scientific), with fluoranthene as reagent anion. 5 µL of sample was loaded on a C4-precolumn (300 µm ID × 5 mm, Thermo Fisher Scientific) at 20 µL/min in 5% acetonitrile, 0.05% TFA. After 5 min desalting, the precolumn was switched online with the analytical C4 nanocolumn (75 µm ID × 15 cm, in-house packed with C4 Reprosil) equilibrated in 95% solvent A (5% acetonitrile, 0.2% formic acid) and 5% solvent B (0.2% formic acid in acetonitrile). Proteins were eluted using the following gradient of solvent B at 300 nL/min flow rate: 5 to 40% during 5 min; 40 to 100% during 33 min. The LTQ-Orbitrap Velos was operated either in single MS or in data-dependent acquisition mode with the XCalibur software. MS scans were acquired in the 500–2000 m/z range with the resolution set to a value of 60,000. For top-down experiments, survey scan MS were acquired in the same way, with a resolution at 30,000. Precursor ions were selected from an inclusion list established thanks to previous MS analyses and were fragmented in ETD and the resulting fragment ions were analyzed in the Orbitrap, at a resolution of 60,000. Isolation width was set at 5 m/z and activation time was fixed to 20 ms for ETD.

### Ethics Statement

Animal studies were performed in accordance with the CNRS guidelines for housing and care of laboratory animals in compliance with the European Community council directive 86/609/EEC guidelines and its implementation in France. The procedures involving mice were reviewed and approved by the relevant Ethics Committee (Comité d’éthique en experimentation animale N°01) and received approval from the French Ministry for High Education and Research after ethical evaluation (number 201508271122464 v2 (APAFIS#1535)). The Etablissement Français du Sang (Toulouse, France) provided human peripheral blood samples under contract 21/PLER/TOU/IPBS01/2013-0042. Experiments with human blood were performed in accordance with the relevant French guidelines and regulations (articles L1243-3, L1243-4 and L1245-2 of the French Public Health Code) and protocols were approved by the French Ministry for High Education and Research (agreement number DC 2012-1715). Informed consents were obtained from the donors before sample collection.

## Electronic supplementary material

Supplementary Dataset 1
