# Supplementary material for: Protein O-mannosylation deficiency increases LprG-associated lipoarabinomannan release by Mycobacterium tuberculosis and enhances the TLR2-associated inflammatory response
Source: Sci Rep. 2017 Aug 11;7:7913. doi: 10.1038/s41598-017-08489-7 (PMC5554173; doi:10.1038/s41598-017-08489-7)
Supplement: Supplementary file 1 — Supplementary Dataset 1 [file 41598_2017_8489_MOESM1_ESM.pdf]

SUPPLEMENTARY INFORMATION

Protein *O*-mannosylation deficiency increases LprG-associated lipoarabinomannan release by *Mycobacterium tuberculosis* and enhances the TLR2-associated inflammatory response.

Henar Alonso[1], Julien Parra[1], Wladimir Malaga[1], Delphine Payros[1], Chia-Fang Liu[1], Céline Berrone[1], Camille Robert[1], Etienne Meunier[1], Odile Burlet-Schiltz[1], Michel Rivière[1]*, Christophe Guilhot[1]*

Supplementary Figure S1: CID-MS/MS spectra of the selected mannoprotein glycopeptides.

Supplementary Figure S2: Construction of *M. tuberculosis* mutants deficient for the production of various *O*-mannosylated proteins.

A) Schematic description of the strategy used to produce and to analyze the various mutants.
B) PCR analyses of the various *M. tuberculosis* mutants using selected couples of primers.

Supplementary Figure S3: Growth of WT, mutant and complemented strains on 7H11 solid medium

A) Serial dilutions of adjusted liquid cultures of the various mutants were spotted onto solid 7H11 OADC medium and incubated at 37°C. The results showed that both the Δ*pmt* and the Δ*lprG* mutants were impaired in their capacity to form colonies.
B) Complementation of the Δ*lprG* mutant**.** Several constructs based on an integrative plasmid harboring either *lprG* or *Rv1410c* alone, or the *lprG-Rv1410c* operon, were transferred in the Δ*lprG* mutant. Complementation was evaluated as the restored capacity to form colonies on 7H11 OADC plate. The results indicated that both *lprG* and *Rv1410c* were required for complementation of the Δ*lprG* mutant.

Supplementary Figure S4: Growth of the bacterial population in the lung of infected mice and proportion of each mutant in the inoculum mix.

A) Number of cfu in the lung of infected mice after 1, 14 and 28 days of infection. Cfu were evaluated by plating serial dilution of lung homogenates on 7H11 OADC plates. The result for each mouse is indicated by a circle.
B) Relative representation of each mutant and WT strain in the inoculum used to infect mice. Total DNA was extracted from the assembled mix used to prepare the inoculum for infection

of the mice. The quantity of each mutant was evaluated by qPCR using primers specific for the tags. The amount of each mutant relative to H37Rv is plotted.

Supplementary Figure S5: Impact of LprG *O*-mannosylation on the release of the LprG/LAM complex in *M. smegmatis* Δ*pmt* or complemented with *pmt* from *M. tuberculosis.*

Western-blot analyses of LAM associated with LprG purified from the *M. smegmatis* Δ*pmt* mutant and the strain complemented with *pmt* from *M. tuberculosis*. Similar amounts of LprG-His protein purified from bacteria or culture medium were separated by SDS-PAGE. After transfer, the LAM or LprG-His molecules were revealed using anti-LAM or anti-His antibodies. Blots are representative of at least three independent experiments.

Supplementary Figure S6: Partial top down ETD fragmentation spectrum of the 22,541 Da molecular mass ion precursor allowing the localization of the unique hexose on the T231.

Peptide sequence of the precursor molecular ion reporting the detected fragment ions. For clarity, the observed ions are numbered according the sequence of the parent molecular ion that starts from the K31 of the gene-encoded sequence.

Supplementary Figure S7: Growth on 7H11 plates of the *M. tuberculosis* recombinant strains producing WT LprG or LprG T231A.

Serial dilutions of adjusted liquid cultures of various WT, mutant, and complemented strains were spotted on solid 7H11 OADC medium and incubated at 37°C. The results show that the capacity to form colonies on solid medium was restored in the strains producing either the LprG WT or the LprG T231A proteins.

Supplementary Figure S8: Growth of WT or *M. tuberculosis* mutants in human phagocytes and TNF-α and IL-10 production by infected cells.

A) Quantification of intracellular bacterial load in hMDM infected with WT, Δ*pmt*, Δ*lprG* or complemented strains. For each time point, 50 infected cells were counted and distributed in three groups: cells containing 1-3 bacteria, 4-8 bacteria or more than 8 bacteria. The plotted results are representative of two independent experiments. They indicate that the capacity of both the Δ*pmt* and Δ*lprG* to multiply within hMDM was attenuated and that the Δ*lprG* phenotype was complemented upon expression of either the *lprG* or the *lprG** genes.

B) Inflammatory response of THP-1 infected with WT, Δ*pmt* or complemented strains. Data are representative of two independent experiments performed in duplicate.

Supplementary Figure S9: NK-κB activation in HEK-TLR2 cells incubated with LprG/LAM complexes purified from bacterial cells or culture medium of *M. smegmatis* Δ*pmt* or *M. smegmatis* Δ*pmt* complemented with *pmt* from *M. tuberculosis*

HEK-TLR2 cells were incubated with 50 ng/ml or 10 ng/ml LprG-His and co-purified LAM and SEAP activity was quantified after 24h. PBS 1x or PAM_3CSK_4 (10 ng/ml) were used as negative and positive controls respectively. Data are presented as the mean +/- SD of at least five independent experiments. ** $P < 0.01$; *$P < 0.05$.

Supplementary Figure S10: Production of TNF-α by bone marrow-derived macrophages from WT, TLR2 -/-, or MyD88-/- mice incubated with LprG/LAM complexes purified from bacterial cells or culture medium of *M. smegmatis* Δ*pmt* or *M. smegmatis* Δ*pmt* complemented with *pmt* from *M. tuberculosis*

BMDM were incubated with 100 ng/ml of LprG-His and co-purified LAM and TNF-α was quantified after 24h by ELISA. PBS 1x, LPS (100 ng/ml) or PAM_3CSK_4 (100 ng/ml) were used as negative and positive controls. Data are presented as the mean +/- SD of four independent experiments performed in duplicate. *** P< 0.001; **P* < 0.05.

Supplementary Table S1: Primers used for the construction analysis of *M. tuberculosis* mutant strains.

Construction of KO mutants				
Reference strain	Mutant	Primers used for recombineiring system (construction AES)	Primers used for verification	reference
M. tuberculosis H37Rv	H37Rv<br>ΔRv0175c	A: AACATTGGCGACGTCAACGAC<br>B: ATC**TACCAGATGCCAGCTAGCCT**TGTTCTGGGCACGCGTCC<br>C : ATC**AAGTCCAACACGTCACGCT**TCTTCGGGTCCGGATGGC<br>D: GGTTCAAACTTACGACGGGG	Aout: ATTCGGTGTCAGCGGTGG<br>Bout: AATGCCAACAAGCCAGCC<br>Cout: AACAACAATGACGGGTCG<br>Dout: ATAACCACAGCGGATGCGG	This work
	H37Rv<br>ΔRv0315	A: GTTCCTCAGCTCATCCGGC<br>B: ATC**TACCAGATGCCAGCTAGCCT**AAAGCCCACCGGTTGCC<br>C: ATC**AACTTCTTCAAGAGCTGCC**CGGTGTTGAACCTTGCGG<br>D: TATGCCTGGCGGATGATGC	Aout : TTTGGATTCGATCCCGACG<br>Bout : TTCTTGATGGGCGTCCG<br>Cout : AAGACCTCAACGAGCCCATCC<br>Dout : TGCTGATCGGTTCGTTTTCCG	This work
	H37Rv<br>ΔRv0838	A : TATCCCAGGCCTGCAGTGC<br>B: TTC**TACCAGATGCCAGCTAGCCT**AGCCCCACCATGAGCAGACG<br>C: TTC**ATCAGCAGCAAGAACGCAT**TGCAACGCAGGGCGTCAG<br>D: AATCCACCAGCTGGTGAGCC	Aout : TAAGCGTTCCCAGCTCCG<br>Bout : TCGGCGACTCGGACCAACGG<br>Cout : TTCGGTCGATGTGACGTTTGC<br>Dout : TATCGCCAGCCAACCGTCCG	This work
	H37Rv<br>ΔRv1411c<br>(Δ*lprG*)	A: CGAATACCTCGACTTCCCCG<br>B : ATC**TACCAGATGCCAGCTAGCCT**CTTGAGAGCCTTGGTCTGCG<br>C : ATC**ACTCGAGCAGTGCACGTAC**TGGCAATTCCGTCCAGATGACC<br>D : AACCACAGCAGCATTCCGGC	Aout : ACCGACAGCGATGCGAGCG<br>Bout: GGTTGGTGGTGAGATCGC<br>Cout: CGATCATCAACTGGCACAGG<br>Dout: CAATCCGAGGATGTTGTGCC	This work
	H37Rv<br>ΔRv3491	A: TTTCCTGATCCTGTCCCGGC<br>B: TCC**TACCAGATGCCAGCTAGCCT**CAATTCCCAGTGCGACGG<br>C: TCC**ACTACGCCATGTCGTCGTC**TTCACTGCAGCTCATCGGCCC<br>D: AATTGGGTGGATCTCATGCG	Aout: TATGCAGATGCCGTGGCG<br>Bout: AGGAGTAACTGCCATCCG<br>Cout:CGTTCAGCGACGAGACGC<br>Dout: ATCTATGACCAACTCAGCG	This work
	H37Rv<br>km^{R}	TTTTTTTCATGATAATAATGGTTTCTTAGACG<br>AAAAAAAAGCTTCTGTGCTGTTGTACATGTCGATACCAGATGCCAG<br>CTAGCATG	KmFw: GACCATCAAGCATTTTATCCG<br>KmRv: ACCGAGGCAGTTCCATAGG	This work
Kanamycin	K1<br>K2<br>K3<br>K4	GACCATCAAGCATTTTATCC<br>ACCGAGGCAGTTCCATAGG<br>GCCTAGAGCAAGACGTTTCC<br>AATTTAAAAGGATCTAGGTGAAGATCC		This work

Restriction sites are underlined ; Common region and specific target of each mutant used in qPCR analysis are marked in bold.

Supplementary Table S2: Strategy used for the construction of the various complementation plasmids

Complementation strains			
Reference Strain	Complementation	Description	reference
M. tuberculosis H37Rv Δ*lprG*	H37Rv Δ*lprG:: Rv1410c*	Amplification of the *Rv1410c* gene with the primers AAGCAGCTGGTCGAGGAGG and TTTTAAGCTTCTTGGTCGGCACCGGC and insertion between the *Puv*II and *Hind*III sites of pMV361e Hyg integrative plasmid	This work
	H37Rv Δ*lprG:: lprG*	Amplification of the *lprG* gene with the primers TTTTATCAGCTGGACCTCAAACCAG and TGCAGCATAAGCTTGCGCC and insertion between the *Puv*II and *Hind*III sites of pMV361e Hyg integrative plasmid	This work
	H37Rv Δ*lprG:: lprG-Rv1410c*	Amplification of the operon *lprG-Rv1410c* performed with primers TTTTATCAGCTGGACCTCAAACCAG and TTTTAAGCTTATCGCCGACGCGATCTTGGTC and insertion between the *Puv*II and *Hind*III sites of pMV361e Hyg integrative plasmid	This work
	H37Rv Δ*lprG:: lprG-Rv1410c gfp*	Amplification of the operon *lprG-Rv1410c* performed with primers TTTTATCAGCTGGACCTCAAACCAG and TTTTAAGCTTATCGCCGACGCGATCTTGGTC and insertion the *Puv*II and *Hind*III sites of pMV361e Hyg gfp integrative plasmid	This work
	H37Rv Δ*lprG:: lprG*-Rv1410c gfp*	The punctual mutation was introduced by amplification of the whole integrative plasmid pMV361 *lprG-Rv1410c gfp* Hyg with CGAGAAGGTCCAGGTCGCGAAGCCCCCGGTGAGCTGATC GATCAGCTCACCGGGGGCTTCGCGACCTGGACCTTCTCG	This work

Supplementary Table S3: Primers used for the qPCR analysis

Name	Sequence
Rv0175c variable	GTCTTCTCGACCACCACAGAT
Rv0315 variable	GGGCAGCTCTTGAAGAAGTT
Rv0838 variable	ATGCGTTCTTGCTGCTGAT
Rv3491 variable	GACGACGACATGGCGTAGT
Rv1411c variable	AGTACGTGCACTGCTCGAGT
qPCR km Fw	CGATGAGTTTTTCTAACTGTCAGACC
qKmFint	TGATGCGCTGGCAGTGTT
qKmRint (commonR)	CGCGATCGCTGTTAAAAGGA

MS/MS Fragmentation of **DCVAATQAPDAGAMSASMQK**
Found in **RVBD_0175T.1** in **MTBtuberculist_R27**, RVBD_0175T.1|RVBD_0175|Mycobacterium tuberculosis H37Rv (Broad V3) MCE-associated membrane protein (214 aa)

Match to Query 77813: 2170.921448 from(1086.468000,2+) index(78042)
Variable modifications:
C2 : Carbamidomethyl (C)
S17 : HexINL (ST), with neutral losses 162.052823(shown in table), 0.000000
Ions Score: 121 **Expect:** 6.9e-012

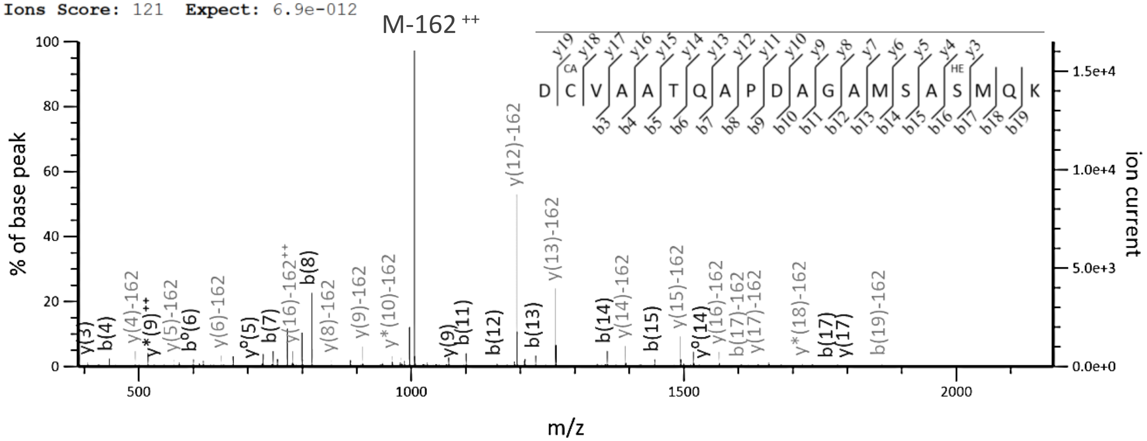

MS/MS Fragmentation of **PAGPTPAPAAPAAATGGLLFHDEFDGPAGSVPDPSK**
Found in **RVBD_0315T.1** in **MTBtuberculist_R27**, RVBD_0315T.1|RVBD_0315|Mycobacterium tuberculosis H37Rv (Broad V3) hypothetical protein (295 aa)

Match to Query 15435: 3544.704495 from(1182.575441,3+) index(2655)
Monoisotopic mass of neutral peptide Mr(calc): 3544.699829
Variable modifications:
S35 : Hex(1)NLI(ST) (ST), with neutral loss 162.052823
Ions Score: 29 **Expect:** 0.0043

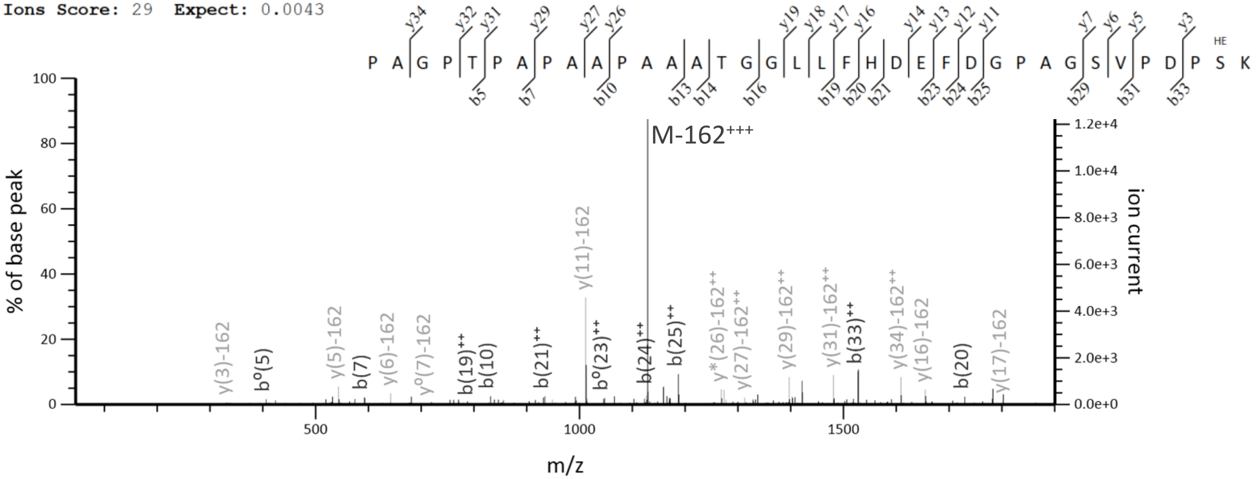

MS/MS Fragmentation of **VQVTKPPVS**
Found in **RVBD_1411cT.1** in **MTBtuberculist_R27**, RVBD_1411cT.1|RVBD_1411c|Mycobacterium tuberculosis H37Rv (Broad V3) lipoprotein lprG (237 aa)

Match to Query 16700: 1115.607248 from(558.810900,2+) index(80564)

Monoisotopic mass of neutral peptide Mr(calc): 1115.607422
Variable modifications:
T4 : HexINL (ST), with neutral losses 0.000000(shown in table), 162.052823
Ions Score: 25 **Expect:** 0.039 (help)

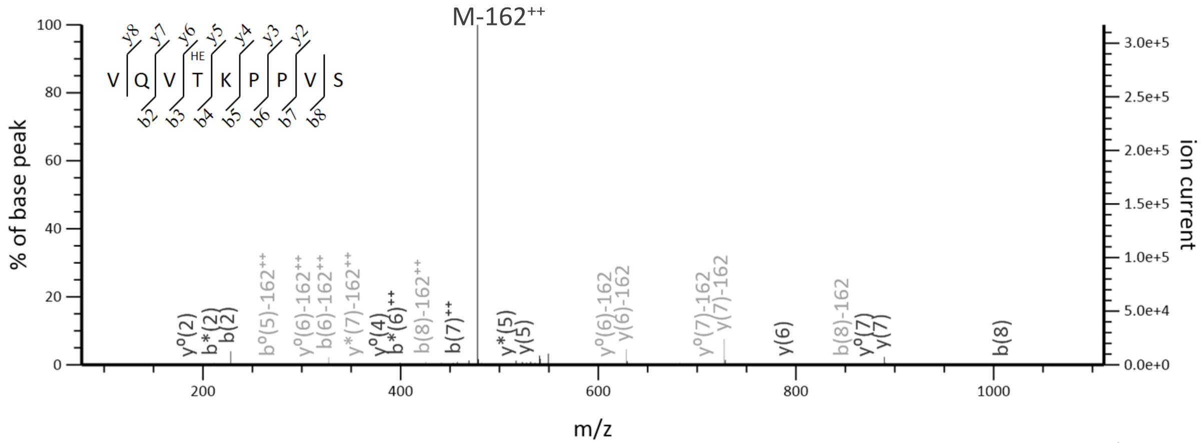

MS/MS Fragmentation of **TTTPSGPVPPVSEAAR**
Found in **RVBD_0838T.1** in **MTBtuberculist_R27**, RVBD_0838T.1|RVBD_0838|Mycobacterium tuberculosis H37Rv (Broad V3) D-alanyl-D-alanine dipeptidase (257 aa)

Match to Query 70844: 1889.908848 from(945.961700,2+) index(55551)

Monoisotopic mass of neutral peptide Mr(calc): 1889.910599
Variable modifications:
S5 : HexINL (ST), with neutral losses 162.052823(shown in table)
S12 : HexINL (ST), with neutral losses 162.052823(shown in table)
Ions Score: 55 **Expect:** 1.4e-005

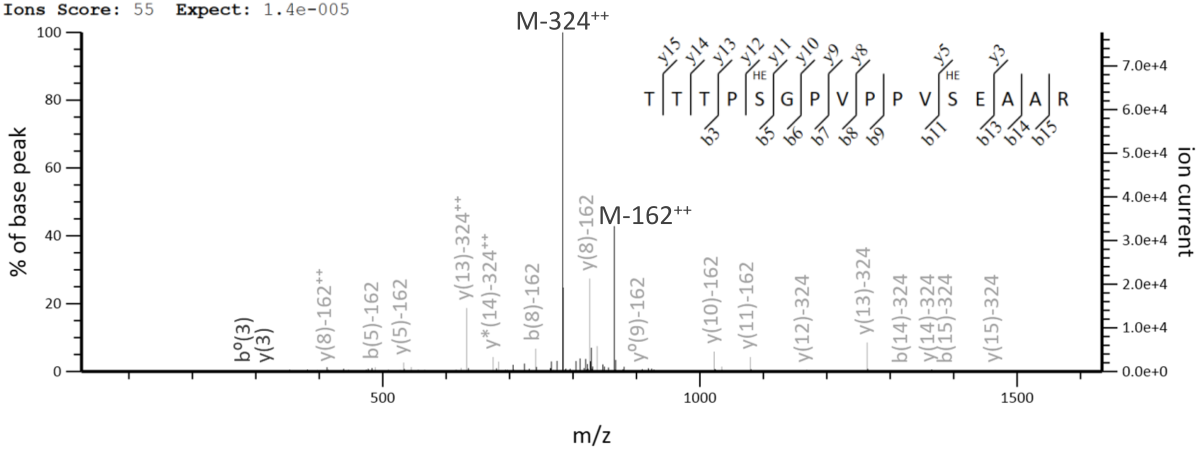

MS/MS Fragmentation of **QPFSLQLIGPPPSPVQR**
Found in **Rv3491_Rv3491** in **MTBtuberculist_R27**, Rv3491_Rv3491

Match to Query 75159: 2022.089448 from(1012.052000,2+) index(76792)

Monoisotopic mass of neutral peptide Mr(calc): 2022.078598
Variable modifications:
S13 : HexINL (ST), with neutral losses 162.052823(shown in table)
Ions Score: 49 **Expect:** 3.4e-005

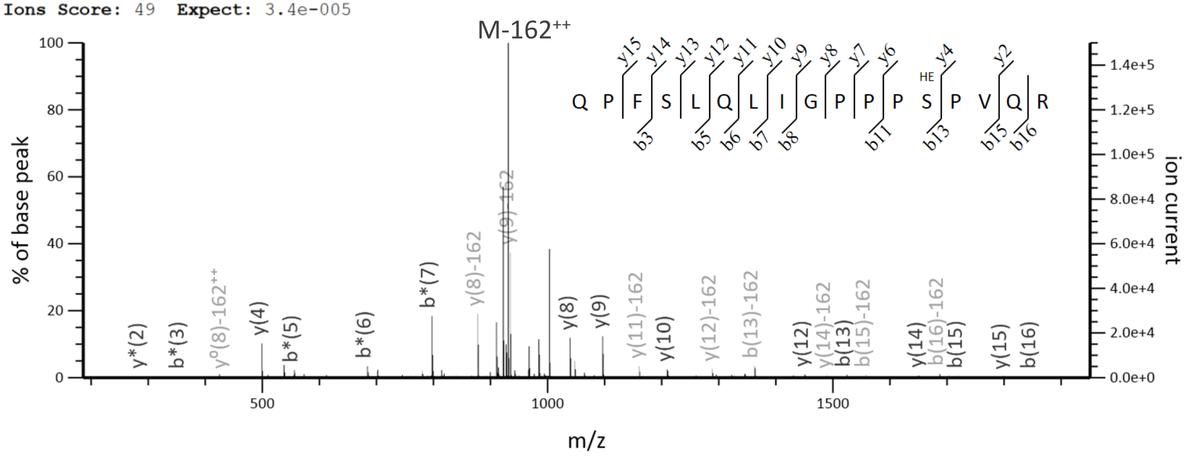

Figure S1

A)

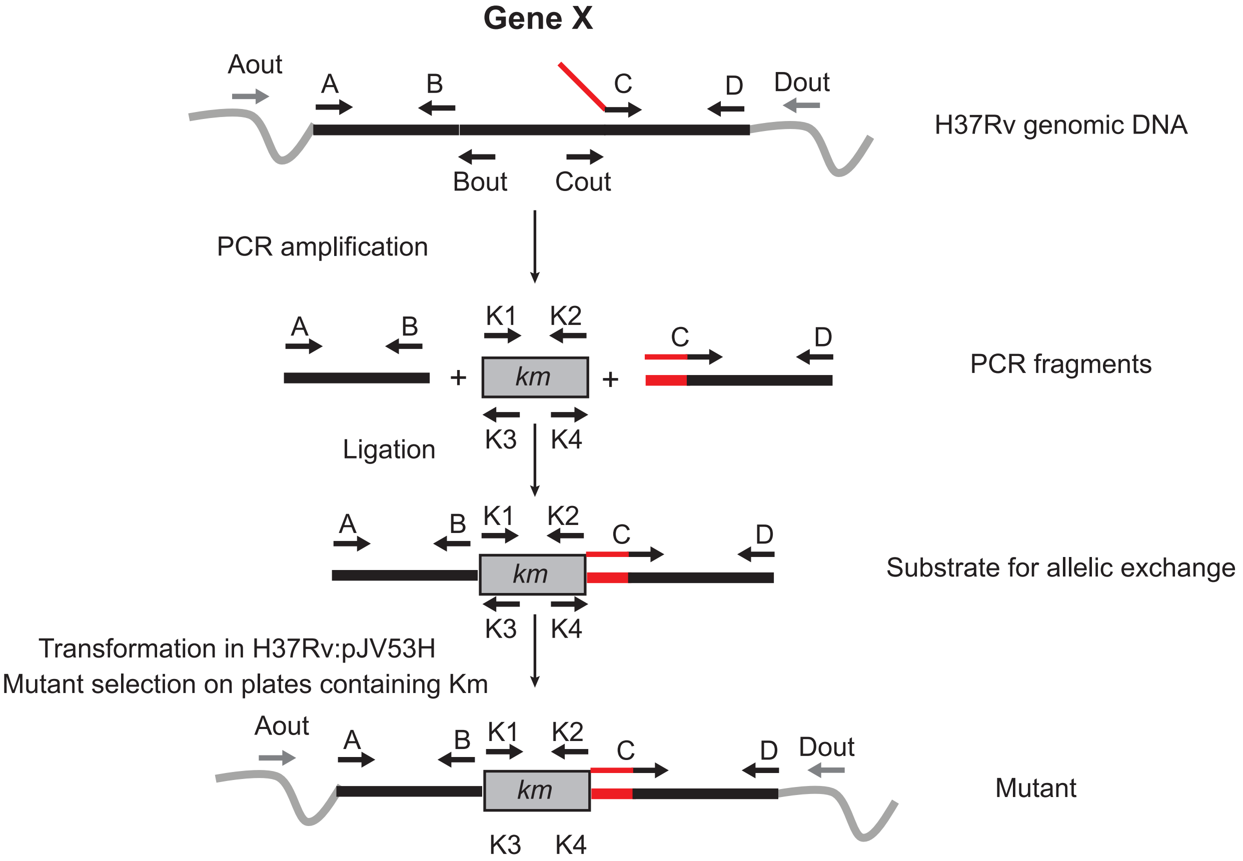
Gene X
Aout
A
B
C
D
Dout
H37Rv genomic DNA
Bout
Cout
PCR amplification
K1
K2
km
K3
K4
PCR fragments
Ligation
Substrate for allelic exchange
Transformation in H37Rv:pJV53H
Mutant selection on plates containing Km
Mutant

B)

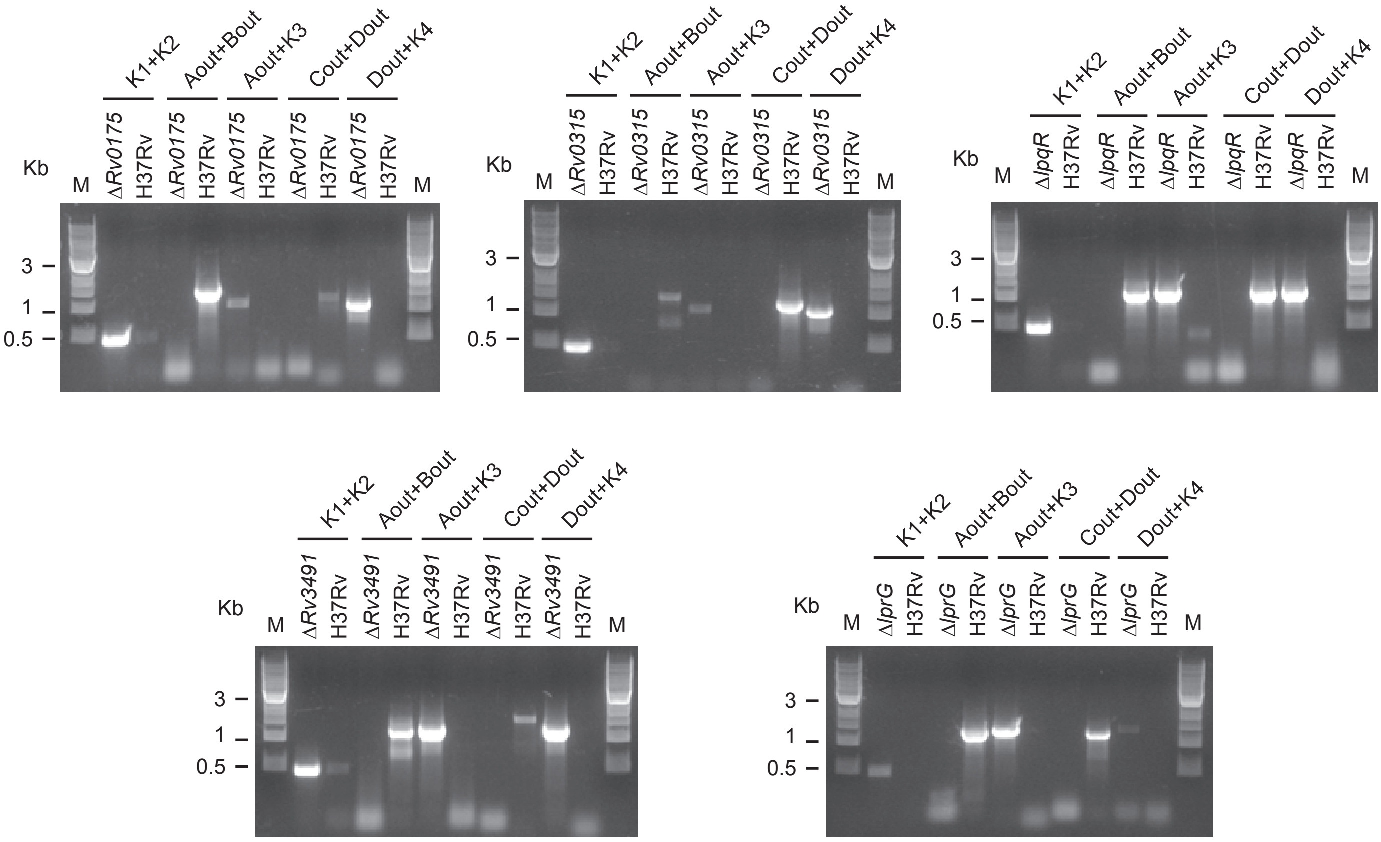
K1+K2
Aout+Bout
Aout+K3
Cout+Dout
Dout+K4
Kb
M
ΔRv0175
H37Rv
ΔRv0315
ΔlpqR
ΔRv3491
ΔlprG
3
1
0.5

Figure S2

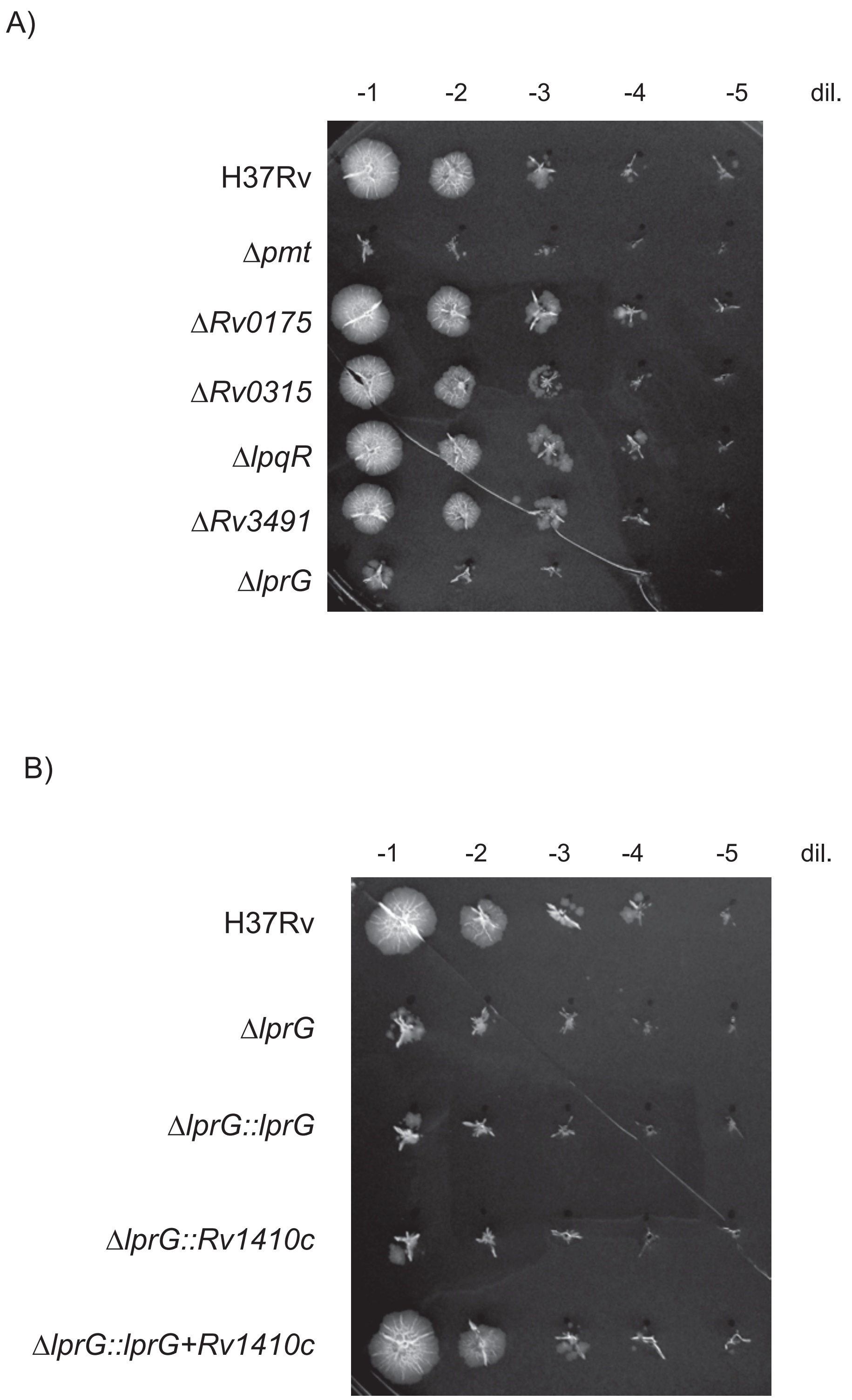
A)
-1
-2
-3
-4
-5
dil.
H37Rv
ΔpmtΔ
ΔRv0175
ΔRv0315
ΔlpqR
ΔRv3491
ΔlprG
B)
-1
-2
-3
-4
-5
dil.
H37Rv
ΔlprG
ΔlprG::lprG
ΔlprG::Rv1410c
ΔlprG::lprG+Rv1410c

Figure S3

A)

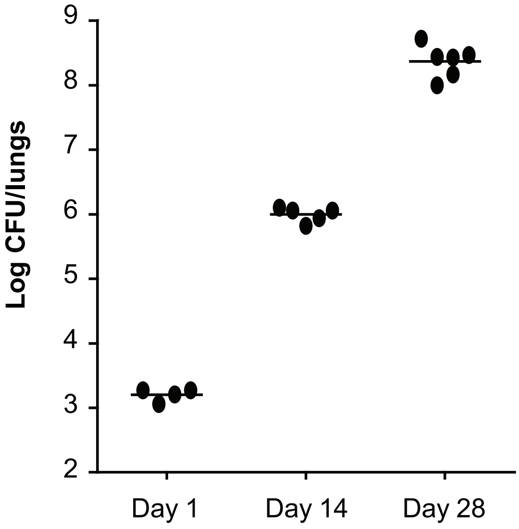
Log CFU/lungs
9
8
7
6
5
4
3
2
Day 1
Day 14
Day 28

B)

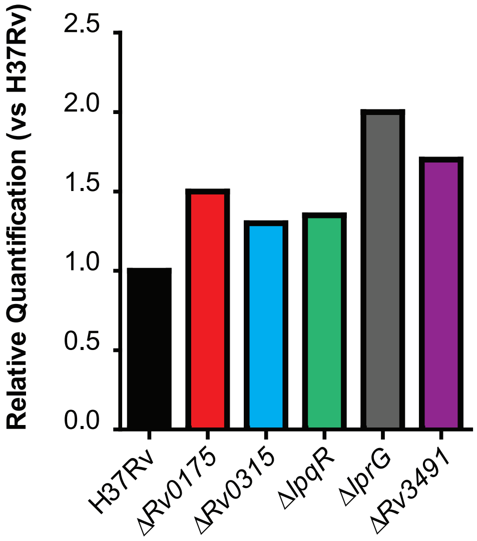
Relative Quantification (vs H37Rv)
2.5
2.0
1.5
1.0
0.5
0.0
H37Rv
ΔRv0175
ΔRv0315
ΔlpqR
ΔlprG
ΔRv3491

Figure S4

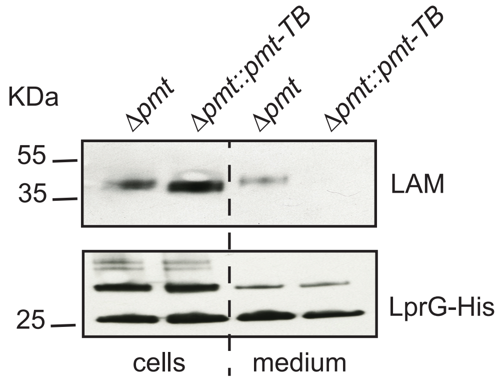
KDa
Δpmt
Δpmt::pmt-TB
Δpmt
Δpmt::pmt-TB
55
35
LAM
LprG-His
25
cells
medium

Figure S5

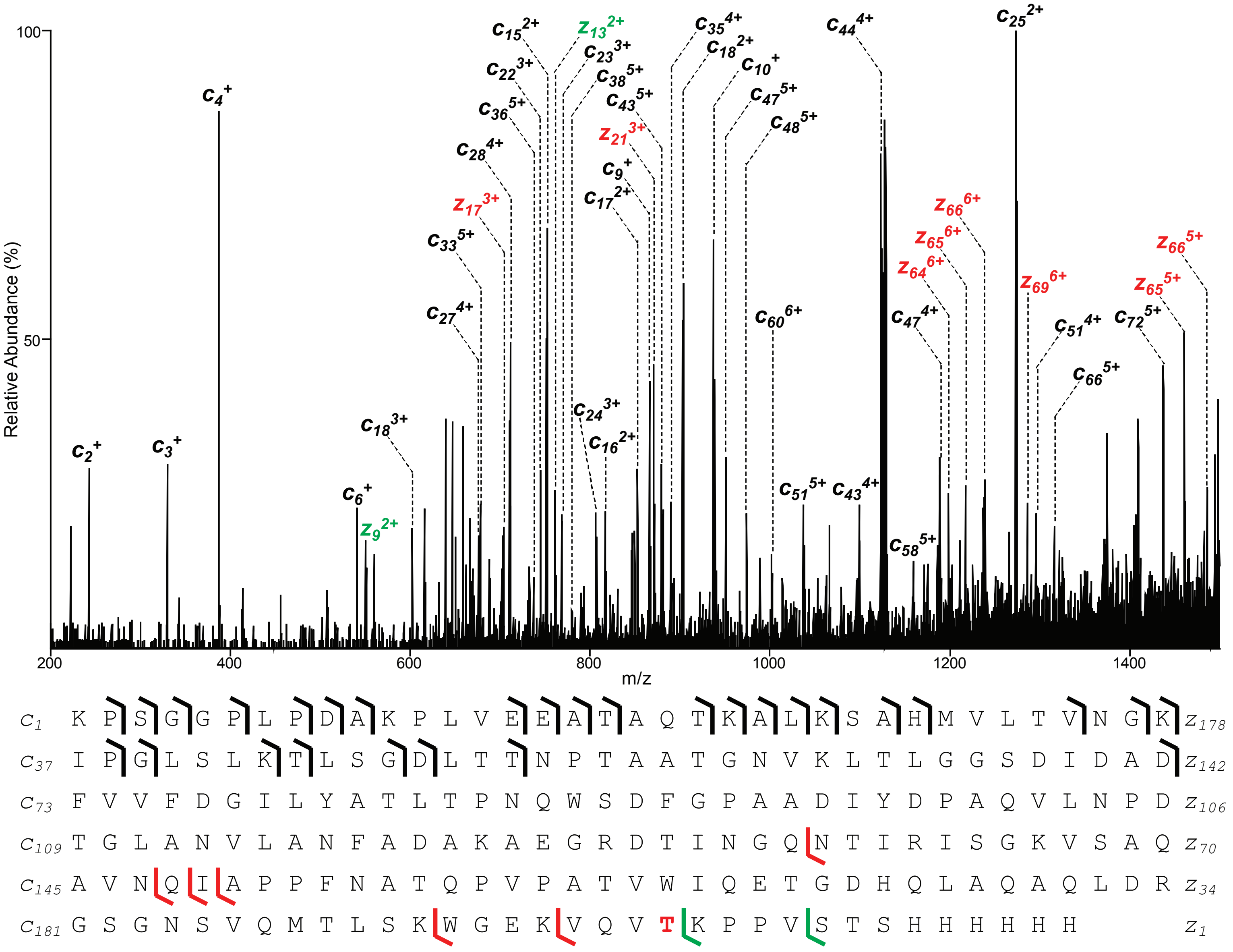

Relative Abundance (%)
100
50
200
400
600
800
1000
1200
1400
m/z
c1 K P S G G P L P D A K P L V E E A T A Q T K A L K S A H M V L T V N G K z178
c37 I P G L S L K T L S G D L T T N P T A A T G N V K L T L G G S D I D A D z142
c73 F V V F D G I L Y A T L T P N Q W S D F G P A A D I Y D P A Q V L N P D z106
c109 T G L A N V L A N F A D A K A E G R D T I N G Q N T I R I S G K V S A Q z70
c145 A V N Q I A P P F N A T Q P V P A T V W I Q E T G D H Q L A Q A Q L D R z34
c181 G S G N S V Q M T L S K W G E K V Q V T K P P V S T S H H H H H H z1

Figure S6

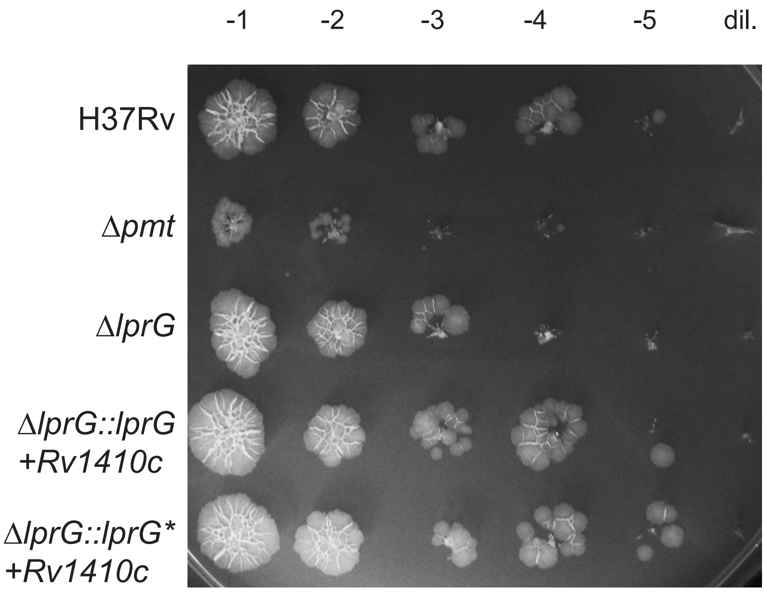
-1
-2
-3
-4
-5
dil.
H37Rv
Δpmt
ΔlprG
ΔlprG::lprG
+Rv1410c
ΔlprG::lprG*
+Rv1410c

Figure S7

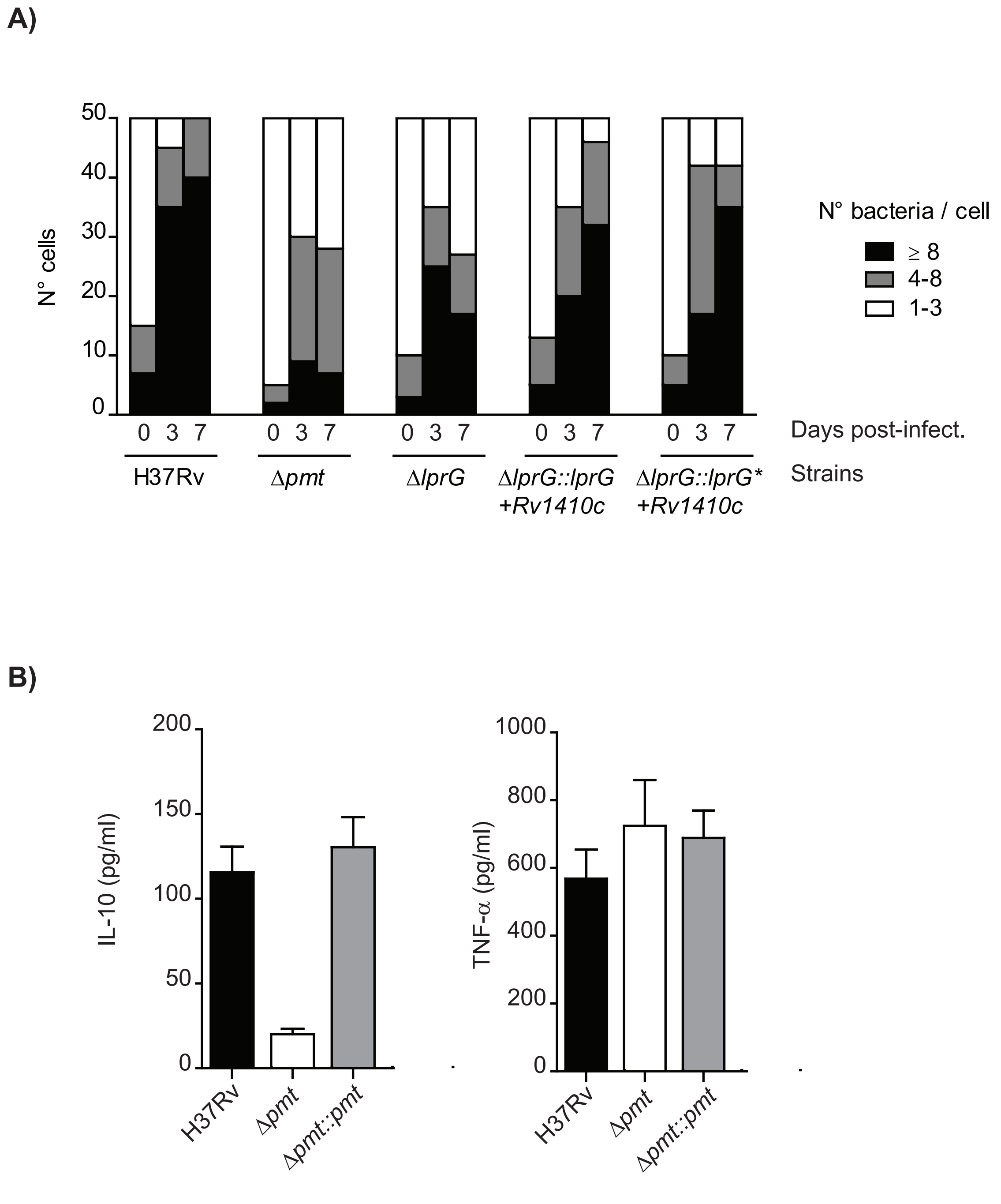
A)
N° cells
50
40
30
20
10
0
0 3 7
Days post-infect.
H37Rv
Δpmt
ΔlprG
ΔlprG::lprG +Rv1410c
ΔlprG::lprG* +Rv1410c
Strains
N° bacteria / cell
≥ 8
4-8
1-3
B)
IL-10 (pg/ml)
200
150
100
50
0
TNF-α (pg/ml)
1000
800
600
400
200
0
H37Rv
Δpmt
Δpmt::pmt

Figure S8

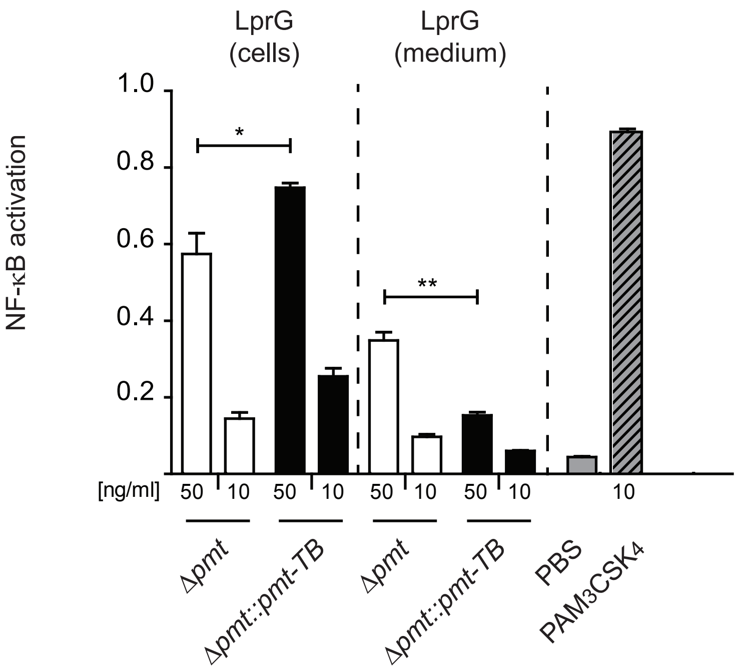
LprG
(cells)
LprG
(medium)
NF-κB activation
1.0
0.8
0.6
0.4
0.2
*
**
[ng/ml]
50
10
50
10
50
10
50
10
10
Δpmt
Δpmt::pmt-TB
Δpmt
Δpmt::pmt-TB
PBS
PAM3CSK4

Figure S9

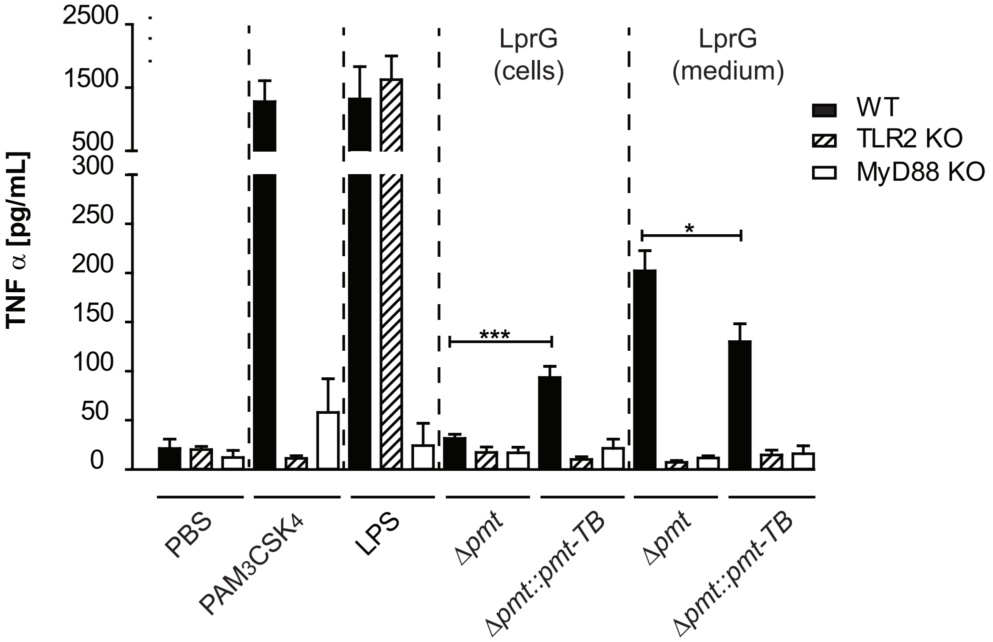

2500
1500
500
300
250
200
150
100
50
0
TNF α [pg/mL]
LprG (cells)
LprG (medium)
WT
TLR2 KO
MyD88 KO
***
*
PBS
PAM3CSK4
LPS
Δpmt
Δpmt::pmt-TB
Δpmt
Δpmt::pmt-TB

Figure S10
